# Personal, community, and societal factors associated with *mukbang* viewing among adolescents: findings from the Korea Youth Risk Behavior Survey

**DOI:** 10.4178/epih.e2025055

**Published:** 2025-09-30

**Authors:** Cynthia Yursun Yoon, Seungha Shin, Haemi Jun, Hyeeun Park, Minseo Kim

**Affiliations:** 1 Department of Food Science and Nutrition, Pusan National University, Busan, Korea; 2 Kimchi Research Institute, Pusan National University, Busan, Korea

**Keywords:** Adolescents, Media, Binge eating, Eating behavior, Korea Youth Risk Behavior Survey

## Abstract

**OBJECTIVES:**

*Mukbang* refers to livestreamed videos in which hosts consume large amounts of energy-dense, nutrient-poor foods while interacting with viewers. *Mukbang* is widely viewed by Korean adolescents and has been associated with adverse health outcomes. To inform efforts to prevent excessive engagement with *mukbang* content among Korean adolescents, this study examined personal, community, and societal factors associated with excessive *mukbang* viewing (≥7 times/wk) and explored gender differences in these associations.

**METHODS:**

Data were drawn from the 2022 Korea Youth Risk Behavior Survey (n=36,990; mean age, 15.1±1.7 years; 48.6% girls; 53.2% attending middle school). Self-reported measures included personal (e.g., perceived health, weight, stress, depression, anxiety), community (e.g., living arrangement), and societal factors (e.g., socioeconomic status) and *mukbang* viewing frequency. Logistic and modified Poisson regression models were used to examine associations with excessive *mukbang* viewing and to evaluate differences by gender.

**RESULTS:**

Intrapersonal factors—namely perceived health, weight, stress, depression, and anxiety—were associated with excessive *mukbang* viewing (adjusted prevalence ratios, 1.18 to 1.44), with more pronounced relationships among girls. A community-level factor—living arrangement—displayed a significant association in boys but not in girls. Boys living on campus had 1.42 times the prevalence of excessive *mukbang* viewing than boys residing with family members (95% confidence interval, 1.08 to 1.88) after adjustment. Further mutual adjustment attenuated estimates toward the null. Societal factors were not significantly associated with excessive *mukbang* viewing among adolescents.

**CONCLUSIONS:**

Personal and community factors were associated with excessive *mukbang* viewing. Future research should investigate the mechanisms underlying these associations.

## GRAPHICAL ABSTRACT

[Fig f2-epih-47-e2025055]

## Key Message

Personal factors such as poor perceived health, stress, depression, and anxiety were strongly related to excessive *mukbang* viewing, particularly among girls, suggesting that emotional distress may drive girls to engage with *mukbang* as a form of coping or personal connection. Among boys, those living on campus were more likely to view *mukbang* excessively than those living with family, indicating that community environment and peer influence may contribute to higher engagement in *mukbang*. Socioeconomic status was not significantly associated with excessive *mukbang* viewing, implying that prevention efforts should address emotional well-being and healthy media use across all adolescents, regardless of the background.

## INTRODUCTION

*Mukbang* refers to livestreamed videos in which hosts consume unusually large quantities of energy-dense, nutrient-poor foods while interacting with viewers in real time [1-6]. While *mukbang* has become a global phenomenon [3], it is particularly prevalent in Korea, particularly among adolescents [6]. Approximately 70.6% of Korean adolescents have been exposed to *mukbang* during the past year, and 13.2% reported viewing *mukbang* 5 or more times per week [6]. This high prevalence among Korean adolescents may reflect Korea’s collectivist culture, which places a strong value on communal eating and social connectedness [7,8]. Within this context, *mukbang* may serve as a digital extension of traditional offline mealtime gatherings and reinforce shared food-related experiences. Additionally, since adolescents are developing their identities and are especially sensitive to the influence of family, peers, and media, *mukbang* videos hosted by influencers—whom adolescents may view as role models—can provide further appeal. This pattern of *mukbang* consumption among Korean adolescents is concerning, as it may normalize excessive intake of energy-dense, nutrient-poor foods, often consumed within a short period (e.g., 2 hours). This type of content may also distort viewers’ perceptions of appropriate portion sizes and promote eating driven by external cues (e.g., watching others) or emotional states (e.g., boredom) rather than internal hunger signals [2,5]. These factors could potentially increase the risk of overeating or binge eating, which in turn can contribute to obesity [9-11].

The substantial prevalence of *mukbang* viewing among Korean adolescents [6] and its potential harmful consequences [9-11] underscore the need to examine factors that may place adolescents at heightened risk of excessive exposure to *mukbang*. This inquiry is theoretically grounded in the compensatory internet use model, which suggests that individuals may turn to online media to partially fulfill unmet emotional and psychological needs [12]. Additionally, the social ecological model [13] provides a conceptual framework to posit that personal, socio-cultural, and environmental factors influence adolescents’ exposure to *mukbang*. For example, personal factors such as anxiety and loneliness may increase emotional distress, leading adolescents to engage with *mukbang* media to cope or seek comfort [1,14,15]. Community-level factors such as living arrangements may also shape adolescents’ engagement in *mukbang*. Living alone or off campus can result in greater social and emotional isolation, which may increase reliance on online media for comfort or companionship [16,17] in managing these feelings. Societal factors, particularly socioeconomic status (SES), might further compound exposure to online or social media and result in excessive exposure to *mukbang*. Adolescents from lower-SES backgrounds may turn to online media, including *mukbang*, for vicarious experiences or emotional support due to limited food access and financial stress [18-21].

Emerging evidence further suggests that media consumption patterns may vary between girls and boys [22,23], reflecting gender differences in motivations for engaging with food-related media [24] and in susceptibility to psychosocial stressors [23]. Studies have documented that personal factors such as emotional loneliness are more commonly experienced by girls than boys [4,25-27], which could increase their likelihood of turning to media as a form of emotional coping. Adolescent girls living alone or off campus may perceive greater social and emotional isolation, potentially heightening their reliance on online media for comfort or companionship [16,17,28]. Societal factors, particularly SES, might also influence *mukbang* viewing in a gender-specific manner. When offline support is limited, girls from lower-SES backgrounds may seek comfort in portrayals of indulgent foods, social interaction, and a sense of inclusion [29]. For boys, economic or social marginalization may make traditional forms of masculine identity feel out of reach, leading them to symbolic performances—such as observing the consumption of large quantities of food—as a form of compensatory identity construction [30]. Understanding how personal, community, and social factors interact, as well as how they may differ by gender, could help identify adolescents most at risk and inform targeted interventions to prevent excessive exposure to *mukbang*.

Building on prior research and theoretical models, this study aims to explore personal, community, and societal factors in relation to excessive *mukbang* viewing (≥7 times/wk) among Korean adolescents and to examine how such factors differ by gender. We hypothesized that personal, community, and societal factors would be associated with excessive *mukbang* viewing among adolescents. Furthermore, given that girls tend to experience more emotional and social loneliness than boys, these factors were expected to display stronger associations in girls.

## MATERIALS AND METHODS

### Study sample and data

The Korea Youth Risk Behavior Survey (KYRBS) is a nationwide repeated cross-sectional survey that assesses the health and health-related behaviors of Korean adolescents (ages 12 to 18 years) [31]. Selected students voluntarily and anonymously complete a self-administered online questionnaire [31], with de-identified raw data analyzed for this study. Detailed information about the KYRBS is available elsewhere [31].

**Figure 1.**
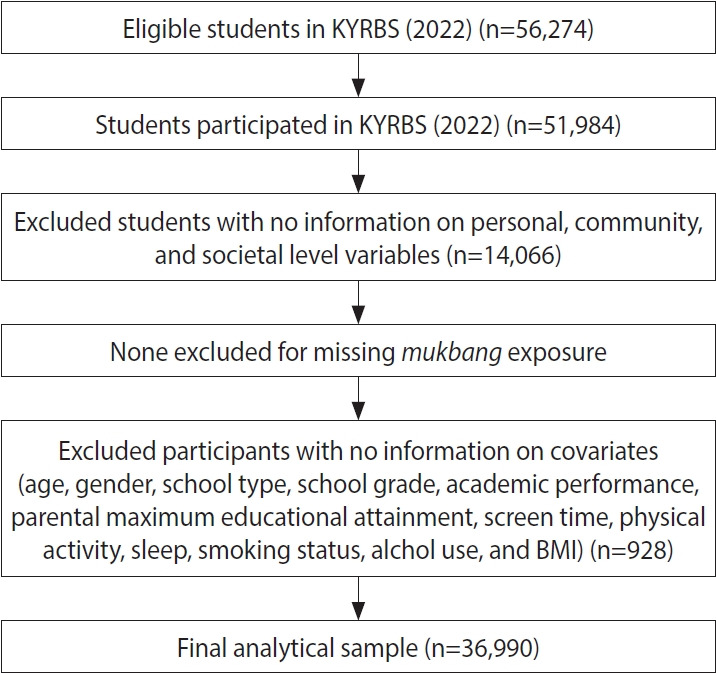
Flow chart. KYRBS, Korea Youth Risk Behavior Survey; BMI, body mass index.

The 18th wave of the KYRBS was administered in 2022, targeting 56,274 adolescents; 51,984 responded (response rate, 92.4%). Non-participation (n=4,290) occurred mainly because of teachers’ heavy workloads or limited access to computer labs. An additional 14,994 participants were excluded due to missing data, yielding an analytic sample of 36,990 adolescents (19,579 middle school students and 17,311 high school students). A detailed flow diagram of participants is presented in Figure 1.

### Measures

Independent variables included self-reported personal, community, and societal factors. The variables, the text of the questions, and the response options are provided in Table 1.

The dependent variable was excessive *mukbang* viewing, defined as ≥7 times/wk to capture daily or more frequent engagement and to identify adolescents with the most intensive viewing patterns. This cutoff corresponds to approximately the top 10% of viewers in this study and aligns conceptually with thresholds used to define excessive media use in the digital health literature (e.g., ≥3 hr/day in one study, which also reflected the top 10%) [32]. The questions and response options are detailed in Table 1.

Covariates included age, gender, school type, school grade, academic performance, parental highest educational attainment, screen time, physical activity, sleep, smoking status, alcohol use, and body mass index (BMI).

### Statistical analysis

Data were analyzed using SAS version 9.4 (SAS Institute Inc., Cary, NC, USA). Survey weights were applied according to the KYRBS sampling design. Descriptive statistics were used to characterize respondents. Both overall and gender-stratified analyses were conducted. Although the formal interaction terms between gender and most independent variables did not indicate statistical significance (p for interaction >0.20), gender-stratified results are presented based on theoretical considerations and prior research suggesting gender-specific differences in *mukbang* and eating-related behaviors. Logistic regression was used to estimate the predicted probability (PP) of excessive *mukbang* viewing as a function of multilevel factors [33]. Modified Poisson regression was used to estimate prevalence ratios (PRs) and 95% confidence intervals for excessive exposure to *mukbang* by factor. For the non-stratified analyses, all models were adjusted for age, gender, school type, school grade, academic performance, parental highest educational attainment, screen time, physical activity, sleep, smoking status, alcohol use, and BMI. For gender-stratified analyses, models were adjusted for all covariates except gender. The final model was mutually adjusted for all independent variables.

### Ethics statement

The KYRBS received Institutional Review Board approval from the Korea Disease Control and Prevention Agency (No. 117058) and adhered to the principles of the Declaration of Helsinki. Students were enrolled after they expressed interest, provided written informed consent, and were assured of their right to withdraw at any time.

## RESULTS

### General characteristics of study participants

The analytic sample comprised 36,990 students in 2022; 52.9% were middle school students. The mean age was 15.1±1.7 years, and 48.6% were girls (Table 2).

### Predicted probabilities and prevalence ratios of excessive *mukbang* viewing by personal factors

#### Overall adolescents

After adjustment (Table 3, model 1), the PP of excessive *mukbang* viewing was highest among adolescents who perceived their health as extremely unhealthy (PP, 12.2%), followed by those with obesity (PP, 11.9%), severe anxiety (PP, 10.1%), depression (PP, 9.1%), high loneliness (PP, 9.1%), and high perceived stress (PP, 9.0%). Adolescents who perceived their health as extremely unhealthy had 1.46 times the prevalence of excessive *mukbang* viewing compared with those who perceived their health as extremely healthy (Table 4, model 1). Participants with obesity had 1.68 times the prevalence compared to those who perceived themselves as extremely underweight; those with severe anxiety had 1.44 times the prevalence of those with minimal anxiety (Table 4, model 1). Participants with depression had 1.31 times the prevalence compared to those without depression, those with high loneliness had 1.29 times the prevalence compared to those with low loneliness, and those with high perceived stress had 1.33 times the prevalence relative to those with low perceived stress (Table 4, model 1). These associations were attenuated after mutual adjustment, but the leading personal factors remained consistent (Tables 3 and 4, model 2).

#### Girls

Among girls, the PP of excessive *mukbang* viewing was highest among those who perceived themselves as having obesity (PP, 12.4%) (Table 3, model 1). After adjustment, this group had 1.52 times the prevalence of excessive *mukbang* viewing compared with those who perceived themselves as extremely underweight (Table 4, model 1). Associations were attenuated after mutual adjustment for independent variables (Tables 3 and 4, model 2).

High levels of anxiety were also related to excessive *mukbang* viewing; the PP was 11.7% among those who self-reported severe anxiety (Table 3, model 1), corresponding to 1.48 times the prevalence compared with those reporting minimal anxiety (Table 4, model 1). Other personal factors associated with excessive *mukbang* viewing included moderate or high loneliness (PP, 10.5%), depression (PP, 10.3%), high perceived stress (PP, 10.2%), and perceiving oneself as extremely unhealthy (PP, 10.0%) (Table 3, model 1). PRs relative to the respective reference groups after adjustment are presented in Table 4, model 1. Associations were attenuated toward the null after further adjustment for other independent variables.

#### Boys

Among personal factors, perceiving one’s health as extremely unhealthy emerged as the factor most strongly associated with excessive *mukbang* viewing (PP, 13.0%) (Table 3, model 1). Boys with this perception had 1.74 times the prevalence of those who perceived their health as extremely healthy (Table 4, model 1). Other personal factors related to excessive *mukbang* viewing included perceived obesity (PP, 11.3%), followed by severe anxiety (PP, 8.5%), depression (PP, 8.0%), high loneliness (PP, 7.7%), and high stress (PP, 7.9%) (Table 3, model 1). PRs for these factors, compared with the reference groups, are presented in Table 4, model 1. Little to no change was observed after further mutual adjustment for the independent variables (Tables 3 and 4, model 2).

### Predicted probabilities and prevalence ratios of excessive *mukbang* viewing by community factors

#### Overall adolescents

The PP of excessive *mukbang* viewing was 7.3% among those who had received nutrition education in the past 12 months and 7.7% among those who had not. PPs of excessive *mukbang* viewing varied from 6.3% to 9.8% by living arrangement; however, the probability of excessive *mukbang* viewing did not differ significantly by living situation (Table 3, model 1). These findings remained largely unchanged after further mutual adjustment (Table 3, model 2).

#### Girls

Regarding community-level factors, adolescents living off campus had the highest prevalence of excessive *mukbang* viewing (PP, 13.5%); however, the prevalence of excessive viewing did not differ significantly by living arrangement. In addition, the PP of excessive *mukbang* viewing was similar between those who had not received nutrition education in the past 12 months (PP, 9.0%) and those who had (PP, 8.4%) (Table 3, model 1). Little to no change was observed after further adjustment for other independent variables (Tables 3 and 4, model 2).

#### Boys

The PP of excessive *mukbang* viewing was 9.3% among those who lived on campus, followed by those who lived off campus (7.2%), with family members (6.4%), and with relatives (5.4%) (Table 3, model 1). After adjustment, those who lived on campus had 1.42 times the prevalence of excessive *mukbang* viewing compared with those who lived with family members (Table 4, model 1). No significant difference in excessive *mukbang* viewing was observed between boys who had not received nutrition education in the past 12 months and those who had (Tables 3 and 4, model 1). No significant changes were observed after mutual adjustment (Tables 3 and 4, model 2).

### Predicted probabilities and prevalence ratios of excessive *mukbang* viewing by societal factors

#### Overall adolescents

The PP of excessive *mukbang* viewing ranged from 7.2% among adolescents with medium SES to 9.9% among those with high SES (Table 3, model 1). However, the prevalence of excessive *mukbang* viewing did not differ significantly by SES (Table 4, model 1). These results remained consistent after further mutual adjustment (Tables 3 and 4, model 2).

#### Girls

The PP of excessive *mukbang* viewing was 10.1% among adolescents who self-reported their family’s SES as low, followed by medium (PP, 8.5%) and high (PP, 8.6%) (Table 3, model 1). However, the PRs of excessive *mukbang* viewing in the medium-SES or low-SES groups did not differ significantly compared with those in the high-SES group (Table 4, model 1). No significant differences were observed after further mutual adjustment (Tables 3 and 4, model 2).

#### Boys

The PP of excessive *mukbang* viewing among boys with low SES was 8.7%, corresponding to 1.15 times the prevalence among those with high SES (Table 3, model 1). Little to no difference was observed after further adjustment.

### Sensitivity analyses

Sensitivity analyses were conducted to assess the robustness of the findings across alternative cutoffs for defining excessive *mukbang* viewing (≥1, ≥3, or ≥5 times/wk). The results of excessive *mukbang* viewing (≥1 times/week) are reported in Supplementary Materials 1 and 2; results of excessive *mukbang* viewing (≥3 times/wk) are reported in Supplementary Materials 3 and 4; results of excessive *mukbang* viewing (≥5 times/wk) are reported in Supplementary Materials 5 and 6 respectively.

## DISCUSSION

The overarching aim of this study was to examine gender differences in personal, community, and societal factors related to excessive *mukbang* viewing (≥7 times/wk) among Korean adolescents. We first discuss overall findings regarding multilevel factors related to *mukbang* among Korean adolescents and then present gender-stratified findings, given theoretical and empirical evidence suggesting gender-specific differences in factors related to *mukbang*, despite non-significant interaction terms.

Overall, several personal factors were associated with excessive *mukbang* viewing (≥7 times/wk), including perceiving oneself as extremely unhealthy, perceiving oneself as having obesity, high perceived stress, high loneliness, depression, and severe anxiety. Notably, loneliness and anxiety were more strongly related to excessive *mukbang* viewing in girls than in boys. These findings—showing that personal factors such as high loneliness and anxiety are associated with excessive *mukbang* viewing, especially among girls—partially align with prior studies documenting links between anxiety-related stress and binge-eating behaviors [34] used to soothe negative emotions [35,36]. Additionally, given that *mukbang* is a contemporary social trend among Korean adolescents, avoiding this trend may amplify fear of missing out, reinforcing repeated exposure to *mukbang* in pursuit of belonging to a community [1,37]. This effect may be particularly pronounced among girls, whose smaller, more intimate friendship groups can heighten peer influence [38], thus amplifying behaviors, norms, and coping strategies and increasing sensitivity to social trends. Social modeling [39] may further reinforce *mukbang* viewing, as frequent exposure within peer networks normalizes the behavior. The interplay between personal vulnerability and the socially interactive nature of *mukbang* content may reflect adolescents’ desire for connection. The direct engagement of hosts with viewers during *mukbang*, along with real-time chat features, potentially fosters a sense of community [36,40-42]. Considering Korea’s collectivist culture [7]—characterized by conformity [8], uniformity, and a high value placed on communal eating—*mukbang* may be especially compelling as a socially endorsed activity, offering adolescents, particularly girls a sense of social participation and belonging that may help fulfill their emotional needs.

Although no specific community-level factors were related to excessive *mukbang* viewing among adolescents overall, gender-stratified analyses revealed that boys living on campus were more likely to engage in excessive viewing, whereas no such association was observed in girls. This finding, together with the observation that boys who described their health as extremely unhealthy, perceived themselves to have obesity, or reported high perceived stress also exhibited greater *mukbang* viewing, suggests that campus-specific stressors may play a significant role for boys. Such stressors may include academic pressure, the need to fit in, and heightened peer influence [43], which can lead to increased media consumption as a means of alleviating stress. Limited access to supportive offline coping outlets and a tendency toward sedentary routines—particularly among those living in dormitories—may further contribute to excessive *mukbang* viewing. These factors might reduce motivation for physical activity and encourage obesogenic behaviors. For some boys, *mukbang* may serve as a form of emotional regulation [15] or social substitution, reinforced by peer norms that normalize or encourage such viewing as a shared activity.

The comparable prevalence of *mukbang* across socioeconomic strata, with no significant differences by SES or gender, highlights the need for inclusive approaches that target all adolescents, regardless of background, to raise awareness and prevent excessive *mukbang* viewing, given its associations with adverse health outcomes [9-11].

This study has several strengths. First, it used a nationally representative survey of Korean middle school and high school students in 2022, supporting the generalizability of the results to Korean adolescents. Second, it assessed a wide range of personal, community, and societal factors, enabling a broad examination of their relations to *mukbang* among adolescents.

However, this study also has several limitations. First and foremost, although a nationally representative dataset was used, applying the findings to populations outside adolescents or beyond Korea may be challenging. Additionally, the cross-sectional design precludes the establishment of causal relationships. For instance, although the factors assessed in this study were assumed to be predictors of excessive *mukbang* viewing, reverse causation or a feedback loop may exist given the cross-sectional nature of the design. In other words, excessive *mukbang* viewing might contribute to greater depressive symptoms, heightened anxiety, or poorer self-rated health and weight perception. Individuals experiencing depressive symptoms may also be more likely to engage in excessive *mukbang* viewing as a coping mechanism, which in turn may reinforce or exacerbate their psychological distress over time, creating a potential feedback loop between emotional states and *mukbang* media consumption. Thus, longitudinal studies are also needed to determine whether these personal, community, and societal factors have short-term effects that persist only through adolescence or whether they influence exposure to *mukbang* into emerging adulthood. Furthermore, “excessive exposure” in this study referred to the frequency of viewing within a week rather than the amount of time (i.e., duration) spent watching such content. Future research should explore how personal, community, and societal factors relate to both the frequency and the amount of time spent on such media. Additionally, because the exposure timeframe was limited to the past 12 months, adolescents who were excessively exposed prior to this period may not have been captured, and the measure does not distinguish between acute and chronic exposure.

Moreover, this study relies exclusively on self-reported exposure, outcome, and covariate data, introducing the potential for misclassification bias. Underreporting of *mukbang* viewing and psychological symptoms (e.g., depression, anxiety) may occur due to recall difficulties or social desirability, whereas some variables, such as perceived health, might be overreported. SES may also be inaccurately reported if adolescents conflate their current and aspirational circumstances. These errors are likely non-differential, which may bias results toward the null and potentially underestimate the true associations. Nevertheless, differential misclassification cannot be entirely ruled out; for instance, adolescents with greater psychological distress might report *mukbang* viewing or health characteristics differently, which could either inflate or attenuate observed associations. These limitations should be considered when interpreting the findings of this study. Furthermore, the results may have been influenced by unmeasured residual confounding variables. Lastly, we conducted gender-stratified analyses to explore potential differences in associations with excessive *mukbang* viewing. Although conceptually justified based on prior evidence of gender differences in media use, food attitudes, and emotional coping, the interaction terms for gender-specific associations were not statistically significant for most variables, and confidence intervals for PRs frequently overlapped between girls and boys. Therefore, our findings should be considered exploratory and hypothesis-generating rather than conclusive, and the observed patterns should be interpreted with caution.

The findings of this study have important implications for researchers and clinicians. For researchers, the associations between personal factors related to emotion—such as loneliness, depression, and anxiety—and excessive *mukbang* viewing highlight the need to examine emotion regulation processes and their role in *mukbang*. Investigating gender differences in these mechanisms is especially warranted. Longitudinal studies are also needed to track how personal, community, and societal influences on *mukbang* viewing evolve over time.

For clinicians, the link between emotion-related personal factors and excessive *mukbang* viewing points to a subgroup of adolescents who may be particularly vulnerable. Interventions should focus on improving self-esteem, promoting positive body image, and developing healthy coping strategies to reduce reliance on *mukbang* as a form of emotional avoidance or regulation.

## Figures and Tables

**Figure f2-epih-47-e2025055:**
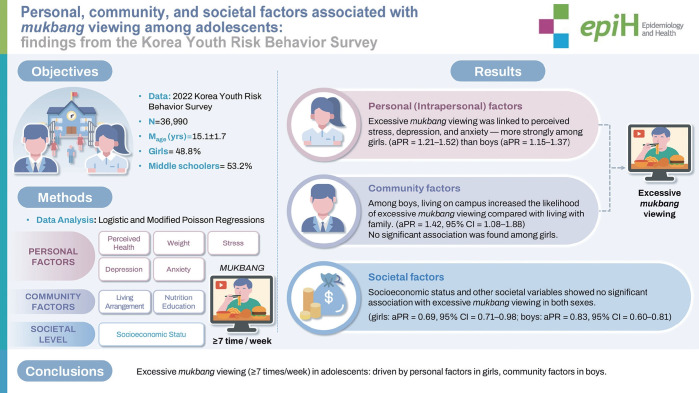


**Table 1.** Questions, response options, and cutoff points for independent, dependent, and moderator variables

Variables	Questions	Response options	Cutoff points
Independent variables			
Perceived health	How would you describe your health status?	Very healthy	
		Slightly healthy	
		Neither healthy nor unhealthy	
		Slightly unhealthy	
		Extremely unhealthy	
Perceived weight	How would you describe your weight?	Very underweight	
		Slightly underweight	
		Normal weight	
		Overweight	
		Obesity	
Perceived stress	How much stress do you normally feel?	I feel a lot of stress	Low: I do not feel much stress or I do not feel stress at all
		I feel a little stress	
		I do not feel much stress	Moderate: I feel a little stress
		I do not feel stress at all	High: I feel a lot of stress
Loneliness	In the past 12 months, how often have you felt lonely?	I felt lonely all the time	Low: I rarely felt lonely or I did not feel lonely at all
		I felt lonely often	
		I felt lonely sometimes	Moderate: I felt lonely sometimes
		I rarely felt lonely	High: I felt lonely all the time or I felt lonely often
		I did not feel lonely at all	
Depression	Have you ever felt so sad or hopeless that it interrupted your daily life for more than 2 weeks in the past 12 months?	Yes	
		No	
Anxiety	Over the past 2 weeks ….	Not at all	0 points: Not at all
	I felt nervous, anxious, or on the edge	Several days	1 point: Several days
	I was unable to stop worrying	More than half of the days	2 points: More than half the days
	I had trouble relaxing	Nearly every day	3 points: Nearly every day
	I was so restless that it was hard to sit still		Total score: 0 to 21 points
	I became easily annoyed or irritable		Normal (minimal anxiety): 0-4 points
	I felt afraid, as if something awful might happen		Mild: 5-9 points
			Moderate: 10-14 points
			Severe: 15-21 points
Nutrition education	Over the past 12 months, did your school provide nutrition education for you?	Yes	
		No	
Living arrangement	Which option best describes your living situation?	Living with immediate family members	
		Living with relatives	
		Living off campus (including with friends)	
		Living at a dormitory or on campus	
Socioeconomic status	What is the financial situation of your household?	High	Low: Low
		Medium to high	Medium: Medium to low, medium, or medium to high
		Medium	
		Medium to low	High: High
		Low	
Dependent variable			
Excessive exposure to binge eating-centric media	Over the past 12 months, how often have you watched *mukbang* (eating broadcast) or cookbang (cooking broadcast)?	Not at all	Excessive exposure to binge eating-centric media: 7 times or more per week
		Less than once per month	
		Once or twice per week	
		Three to four times per week	
		Five to six times per week	
		Daily (7 times or more per week)	
Moderator			
Gender	What was your gender assigned at birth?	Male	
		Female	

**Table 2.** General characteristics of study participants in 2022

Characteristics	Girls (n=17,964)	Boys (n=19,026)	Total (n=36,990)	p-value
Age (yr)^1^	15.1±1.7	15.1±1.7	15.1±1.7	0.01
School level				0.04
Middle school	9,556 (53.8)	10,023 (52.7)	19,679 (53.2)	
High school	8,308 (46.3)	9,003 (47.3)	17,311 (46.8)	
BMI (kg/m^2^)	20.5±3.2	22.2±4.0	21.3±3.7	<0.01
*Mukbang* viewing frequency (times/wk)				<0.01
At least 1	8,461 (47.1)	7,014 (36.9)	15,475 (41.8)	
At least 3	5,026 (28.0)	3,913 (20.6)	8,939 (24.2)	
At least 5	2,680 (14.9)	2,066 (10.9)	4,746 (12.8)	
At least 7	1,641 (9.1)	1,325 (7.0)	2,966 (8.0)	

Values are presented as mean±SD or number (%). SD, standard deviation; BMI, body mass index.

1 Mean age values are rounded to 1 decimal place; The unrounded mean±SD was 15.14±1.74 years for boys and 15.10±1.73 years for girls; The small but statistically significant difference (t(36,988)=2.46, p=0.014) is likely due to the large sample size and is not considered clinically meaningful.

**Table 3.** Predicted probabilities of excessive mukbang viewing by personal, community, and societal factors among Korean adolescents (n=36,990)

Variables			Excessive mukbang viewing (≥7 times/wk)					
			Model 1^1^			Model 2^2^		
			Total	Girls	Boys	Total	Girls	Boys
Personal	Perceived health	Extremely healthy (n=7,624)(girls, n=2,647; boys, n=4,977)	8.3 (7.7, 8.9)	9.3 (8.2, 10.4)	7.3 (6.6, 8.0)	8.6 (7.9, 9.3)	9.8 (8.6, 11.0)	7.5 (6.7, 8.3)
		Slightly healthy (n=16,141)(girls, n=7,958; boys, n=8,183)	6.9 (6.5, 7.3)	8.0 (7.4, 8.6)	6.0 (5.5, 6.5)	6.9 (6.5, 7.3)	8.1 (7.5, 8.7)	5.9 (5.4, 6.4)
		Normal (n=9,582)(girls, n=5,403; boys, n=4,179)	7.6 (7.1, 8.1)	8.9 (8.1, 9.7)	6.3 (5.6, 7.0)	7.3 (6.8, 7.8)	8.6 (7.9, 9.3)	6.0 (5.3, 6.7)
		Slightly unhealthy (n=3,439)(girls, n=1,871; boys, n=1,568)	8.5 (7.6, 9.4)	10.0 (8.6, 11.4)	7.0 (5.7, 8.3)	7.5 (6.6, 8.4)	8.8 (7.5, 10.1)	6.2 (5.0, 7.4)
		Extremely unhealthy (n=204)(girls, n=85; boys, n=119)	12.2 (7.8, 16.6)	10.0 (4.0, 16.0)	13.0 (7.4, 18.9)	10.2 (6.4, 14.0)	8.3 (3.2, 13.4)	10.7 (5.6, 15.8)
	Perceived weight	Extremely underweight (n=1,877)(girls, n=576; boys, n=1,301)	7.0 (5.7, 8.3)	8.0 (7.8, 8.2)	6.1 (4.6, 7.6)	6.8 (5.6, 8.0)	7.9 (5.7, 10.1)	5.9 (4.5, 7.3)
		Slightly underweight (n=8,242)(girls, n=3,517; boys, n=4,725)	6.3 (5.7, 6.9)	7.8 (6.8, 8.8)	5.0 (4.3, 5.7)	6.3 (5.7, 6.9)	7.8 (6.8, 8.8)	4.9 (4.2, 5.6)
		Normal weight (n=13,419)(girls, n=7,408; boys, n=6,011)	7.3 (6.9, 7.7)	8.6 (8.0, 9.2)	6.2 (5.6, 6.8)	7.3 (6.8, 7.8)	8.6 (7.9, 9.3)	6.1 (5.5, 6.7)
		Overweight (n=11,352)(girls, n=5,630; boys, n=5,722)	8.1 (7.5, 8.7)	9.0 (8.1, 9.9)	7.5 (6.6, 8.4)	7.9 (7.3, 8.5)	8.7 (7.8, 9.6)	7.4 (6.5, 8.3)
		Obesity (n=2,100)(girls, n=833; boys, n=1,267)	11.9 (10.0, 13.8)	12.4 (9.5, 15.3)	11.3 (8.8, 13.8)	11.5 (9.7, 13.3)	11.7 (9.0, 14.4)	11.3 (8.8, 13.8)
	Perceived stress	Low (n=6,230)(girls, n=2,324; boys, n=3,906)	6.7 (6.4, 7.3)	8.0 (6.9, 9.1)	5.7 (5.0, 6.4)	7.0 (6.3, 7.7)	8.5 (7.3, 9.7)	5.8 (5.0, 6.6)
		Moderate (n=15,615)(girls, n=7,235; boys, n=8,380)	6.5 (6.1, 6.9)	7.2 (6.6, 7.8)	5.8 (5.0, 6.3)	6.6 (6.2, 7.0)	7.4 (6.8, 8.0)	5.9 (5.4, 6.4)
		High (n=15,145)(girls, n=8,405; boys, n=6,740)	9.0 (8.5, 9.5)	10.2 (9.6, 10.8)	7.9 (7.3, 8.5)	8.5 (8.0, 9.0)	9.6 (8.9, 10.3)	7.5 (6.8, 8.2)
	Loneliness	Low (n=16,859)(girls, n=6,929; boys, n=9,930)	7.0 (6.6, 7.4)	7.9 (7.3, 8.5)	6.2 (5.7, 5.7)	7.5 (7.1, 7.9)	8.5 (7.8, 9.2)	6.5 (6.0, 7.0)
		Moderate (n=13,751)(girls, n=7,228; boys, n=6,523)	7.5 (7.1, 7.9)	8.5 (7.9, 9.1)	6.6 (6.0, 7.2)	7.2 (6.8, 7.6)	8.4 (7.8, 9.0)	6.2 (5.6, 6.8)
		High (n=6,380)(girls, n=3,807; boys, n=2,573)	9.1 (8.4, 9.8)	10.5 (9.5, 11.5)	7.7 (6.7, 8.7)	7.7 (7.0, 8.4)	9.1 (8.1, 10.1)	6.5 (5.5, 7.5)
	Depression	No (n=26,547)(girls, n=12,050; boys, n=14,497)	6.9 (6.6, 7.2)	8.0 (7.5, 8.5)	6.1 (5.7, 6.5)	7.1 (6.8, 7.4)	8.2 (7.7, 8.7)	6.1 (5.7, 6.5)
		Yes (n=10,443)(girls, n=5,914; boys, n=4,529)	9.1 (8.6, 9.6)	10.3 (9.5, 11.1)	8.0 (7.2, 8.8)	8.4 (7.8, 9.0)	9.4 (8.6, 10.2)	7.4 (6.6, 8.2)
	Anxiety	Minimal (n=23,967)(girls, n=10,528; boys, n=13,439)	7.0 (6.7, 7.3)	7.8 (7.3, 8.3)	6.2 (5.8, 6.6)	7.2 (6.8, 7.6)	8.2 (7.6, 8.8)	6.3 (5.9, 6.7)
		Mild (n=8,723)(girls, n=4,814; boys, n=3,909)	8.2 (7.6, 8.8)	9.4 (8.6, 10.2)	7.1 (6.3, 7.9)	7.6 (7.0, 8.2)	8.9 (8.1, 9.7)	6.5 (5.7, 7.3)
		Moderate (n=3,047)(girls, n=1,829; boys, n=1,218)	9.2 (8.2, 10.2)	10.6 (9.2, 12.0)	7.9 (6.4, 9.4)	8.1 (7.1, 9.1)	9.4 (8.0, 10.8)	6.8 (5.4, 8.2)
		Severe (n=1,253)(girls, n=793; boys, n=460)	10.1 (8.5, 11.7)	11.7 (9.5, 13.9)	8.5 (6.1, 11.0)	8.7 (7.2, 10.2)	10.1 (8.0, 12.2)	7.2 (5.0, 9.4)
Community	Nutrition education	No (n=19,707)(girls, n=9,677; boys, n=10,030)	7.7 (7.3, 8.1)	9.0 (8.4, 9.6)	6.6 (6.1, 7.1)	7.6 (7.2, 8.0)	8.9 (8.3, 9.5)	6.5 (6.0, 7.0)
		Yes (n=17,283)(girls, n=8,287; boys, n=8,996)	7.3 (6.9, 7.7)	8.4 (7.8, 9.0)	6.4 (5.9, 6.9)	7.2 (6.8, 7.6)	8.3 (7.7, 8.9)	6.3 (5.8, 6.8)
	Living arrangement	With family members (n=35,515)(girls, n=17,332; boys, n=18,183)	7.5 (7.2, 7.8)	8.7 (8.3, 9.1)	6.4 (6.0, 6.8)	7.4 (7.1, 7.7)	8.6 (8.2,9.0)	6.3 (6.0, 6.6)
		With relatives (n=159)(girls, n=62; boys, n=97)	6.3 (2.6, 10.0)	7.3 (1.0, 13.2)	5.4 (1.1, 9.7)	6.3 (2.6, 10.0)	7.1 (1.0, 13.2)	5.5 (1.2, 9.8)
		Off campus (n=215)(girls, n=84; boys, n=131)	9.8 (6.0, 13.6)	13.5 (6.4, 20.6)	7.2 (3.0, 11.4)	9.4 (5.7, 13.1)	13.1 (6.2, 20.0)	6.8 (2.8, 10.8)
		On campus (n=1,025)(girls, n=451; boys, n=574)	8.6 (6.9, 10.3)	7.8 (5.3, 10.3)	9.3 (6.9, 11.7)	8.5 (6.8, 10.2)	7.7 (5.2, 10.2)	9.1 (6.7, 11.5)
Societal	Socioeconomic status	High (n=4,349)(girls, n=1,818; boys, n=2,531)	9.9 (7.7, 12.1)	8.6 (5.4, 11.8)	10.6 (7.6, 13.6)	9.0 (7.0, 11.0)	7.9 (5.0, 10.8)	9.6 (6.9, 12.3)
		Medium (n=32,131)(girls, n=15,989; boys, n=16,142)	7.2 (6.9, 7.5)	8.5 (8.0, 9.0)	6.1 (5.7, 6.5)	7.2 (6.9, 7.5)	8.4 (7.9, 8.9)	6.0 (5.6, 6.4)
		Low (n=601)(girls, n=248; boys, n=353)	9.5 (8.6, 10.4)	10.1 (8.6, 11.6)	8.7 (7.6, 9.8)	9.4 (8.5, 10.3)	10.1(8.6, 11.6)	8.5 (7.4, 9.6)

Values are presented as predicted probability (95% confidence interval).

1 In model 1, each value represents adjusted for socio-demographic variables (i.e., age, school type, school grade, academic performance, parental highest educational attainment), screen time, physical activity, sleep, smoking status, alcohol use, and body mass index.

2 Model 2 is simultaneously adjusted for all independent variables.

**Table 4.** Prevalence ratios of excessive mukbang viewing by personal, community, and societal factors among Korean adolescents (n=36,990)

Variables			Excessive mukbang viewing (≥7 times/wk)					
			Model 1^1^			Model 2^2^		
			Total	Girls	Boys	Total	Girls	Boys
Personal	Perceived health	Extremely healthy (n=7,624)(girls, n=2,647; boys, n=4,977)	1.00 (reference)	1.00 (reference)	1.00 (reference)	1.00 (reference)	1.00 (reference)	1.00 (reference)
		Slightly healthy (n=16,141)(girls, n=7,958; boys, n=8,183)	0.84 (0.76, 0.93)	0.87 (0.75, 1.01)	0.82 (0.71, 0.93)	0.81 (0.73, 0.89)	0.83 (0.72, 0.96)	0.79 (0.69, 0.90)
		Normal (n=9,582)(girls, n=5,403; boys, n=4,179)	0.92 (0.83, 1.03)	0.97 (0.83, 1.13)	0.87 (0.74, 1.02)	0.84 (0.76, 0.94)	0.87 (0.75, 1.02)	0.80 (0.68, 0.94)
		Slightly unhealthy (n=3,439)(girls, n=1,871; boys, n=1,568)	1.03 (0.90, 1.18)	1.08 (0.89, 1.30)	0.95 (0.77, 1.18)	0.87 (0.76, 1.01)	0.90 (0.74, 1.09)	0.82 (0.66, 1.02)
		Extremely unhealthy (n=204)(girls, n=85; boys, n=119)	1.46 (1.01, 2.15)	1.08 (0.57, 2.03)	1.74 (1.07, 2.85)	1.16 (0.78, 1.71)	0.84 (0.45, 1.60)	1.38 (0.84, 2.27)
	Perceived weight	Extremely underweight (n=1,877)(girls, n=576; boys, n=1,301)	1.00 (reference)	1.00 (reference)	1.00 (reference)	1.00 (reference)	1.00 (reference)	1.00 (reference)
		Slightly underweight (n=8,242)(girls, n=3,517; boys, n=4,725)	0.91 (0.76, 1.10)	0.97 (0.73, 1.30)	0.83 (0.65, 1.07)	0.93 (0.77, 1.12)	0.98 (0.74, 1.32)	0.84 (0.66, 1.08)
		Normal weight (n=13,419)(girls, n=7,408; boys, n=6,011)	1.06 (0.87, 1.28)	1.06 (0.80, 1.42)	1.02 (0.79, 1.32)	1.08 (0.89, 1.31)	1.08 (0.81, 1.45)	1.03 (0.80, 1.33)
		Overweight (n=11,352)(girls, n=5,630; boys, n=5,722)	1.16 (0.93, 1.45)	1.12 (0.81, 1.55)	1.23 (0.91, 1.65)	1.16 (0.93, 1.45)	1.10 (0.80, 1.53)	1.25 (0.92, 1.68)
		Obesity (n=2,100)(girls, n=833; boys, n=1,267)	1.68 (1.27, 2.23)	1.52 (1.01, 2.31)	1.83 (1.23, 2.70)	1.66 (1.24, 2.20)	1.45 (0.96, 2.21)	1.88 (1.27, 2.78)
	Perceived stress	Low (n=6,230)(girls, n=2,324; boys, n=3,906)	1.00 (reference)	1.00 (reference)	1.00 (reference)	1.00 (reference)	1.00 (reference)	1.00 (reference)
		Moderate (n=15,615)(girls, n=7,235; boys, n=8,380)	0.96 (0.86, 1.08)	0.90 (0.76, 1.06)	1.01 (0.87, 1.18)	0.95 (0.85, 1.07)	0.88 (0.74, 1.04)	1.02 (0.87, 1.19)
		High (n=15,145)(girls, n=8,405; boys, n=6,740)	1.33 (1.19, 1.48)	1.27 (1.08, 1.48)	1.37 (1.17, 1.59)	1.22 (1.08, 1.38)	1.12 (0.94, 1.34)	1.30 (1.10, 1.55)
	Loneliness	Low (n=16,859)(girls, n=6,929; boys, n=9,930)	1.00 (reference)	1.00 (reference)	1.00 (reference)	1.00 (reference)	1.00 (reference)	1.00 (reference)
		Moderate (n=13,751)(girls, n=7,228; boys, n=6,523)	1.07 (0.99, 1.16)	1.09 (0.97, 1.22)	1.06 (0.95, 1.20)	0.97 (0.89, 1.06)	0.99 (0.88, 1.12)	0.97 (0.85, 1.10)
		High (n=6,380)(girls, n=3,807; boys, n=2,573)	1.29 (1.17, 1.42)	1.34 (1.17, 1.52)	1.25 (1.07, 1.45)	1.03 (0.92, 1.16)	1.07 (0.92, 1.26)	1.01 (0.84, 1.20)
	Depression	No (n=26,547)(girls, n=12,050; boys, n=14,497)	1.00 (reference)	1.00 (reference)	1.00 (reference)	1.00 (reference)	1.00 (reference)	1.00 (reference)
		Yes (n=10,443)(girls, n=5,914; boys, n=4,529)	1.31 (1.21, 1.41)	1.30 (1.17, 1.43)	1.32 (1.17, 1.49)	1.18 (1.08, 1.29)	1.16 (1.03, 1.30)	1.22 (1.07, 1.40)
	Anxiety	Minimal (n=23,967)(girls, n=10,528; boys, n=13,439)	1.00 (reference)	1.00 (reference)	1.00 (reference)	1.00 (reference)	1.00 (reference)	1.00 (reference)
		Mild (n=8,723)(girls, n=4,814; boys, n=3,909)	1.18 (1.09, 1.29)	1.21 (1.08, 1.35)	1.15 (1.01, 1.32)	1.06 (0.97, 1.17)	1.09 (0.96, 1.23)	1.03 (0.89, 1.19)
		Moderate (n=3,047)(girls, n=1,829; boys, n=1,218)	1.32 (1.17, 1.49)	1.35 (1.16, 1.57)	1.28 (1.05, 1.57)	1.12 (0.98, 1.28)	1.15 (0.96, 1.36)	1.08 (0.871.35)
		Severe (n=1,253)(girls, n=793; boys, n=460)	1.44 (1.22, 1.70)	1.48 (1.20, 1.81)	1.37 (1.01, 1.86)	1.19 (0.98, 1.43)	1.22 (0.97, 1.53)	1.14 (0.82, 1.58)
Community	Nutrition education	No (n=19,707)(girls, n=9,677; boys, n=10,030)	1.00 (reference)	1.00 (reference)	1.00 (reference)	1.00 (reference)	1.00 (reference)	1.00 (reference)
		Yes (n=17,283)(girls, n=8,287; boys, n=8,996)	0.95 (0.88, 1.02)	0.93 (0.84, 1.03)	0.97 (0.87, 1.09)	0.95 (0.88, 1.02)	0.93 (0.84, 1.03)	0.97 (0.87, 1.08)
	Living arrangement	With family members (n=35,515)(girls, n=17,332; boys, n=18,183)	1.00 (reference)	1.00 (reference)	1.00 (reference)	1.00 (reference)	1.00 (reference)	1.00 (reference)
		With relatives (n=159)(girls, n=62; boys, n=97)	0.82 (0.45, 1.49)	0.80 (0.33, 1.93)	0.83 (0.37, 1.85)	0.83 (0.46, 1.50)	0.79 (0.33, 1.92)	0.85 (0.38, 1.90)
		Off campus (n=215)(girls, n=84; boys, n=131)	1.28 (0.85, 1.92)	1.50 (0.86, 2.60)	1.11 (0.61, 2.02)	1.24 (0.83, 1.87)	1.47 (0.85, 2.56)	1.07 (0.59, 1.94)
		On campus (n=1,025)(girls, n=451; boys, n=574)	1.13 (0.92, 1.40)	0.89 (0.64, 1.25)	1.42 (1.08, 1.88)	1.14 (0.92, 1.41)	0.90 (0.64, 1.25)	1.42 (1.08, 1.88)
Societal	Socioeconomic status	High (n=4,349)(girls, n=1,818; boys, n=2,531)	1.00 (reference)	1.00 (reference)	1.00 (reference)	1.00 (reference)	1.00 (reference)	1.00 (reference)
		Medium (n=32,131)(girls, n=15,989; boys, n=16,142)	0.75 (0.67, 0.84)	0.83 (0.71, 0.98)	0.69 (0.60, 0.81)	0.75 (0.67, 0.84)	0.82 (0.70, 0.97)	0.70 (0.60, 0.81)
		Low (n=601)(girls, n=248; boys, n=353)	0.99 (0.77, 1.27)	0.81 (0.54, 1.21)	1.15 (0.83, 1.58)	0.91 (0.70, 1.17)	0.74 (0.49, 1.12)	1.05 (0.76, 1.45)

Values are presented as prevalence ratio (95% confidence interval).

1 In model 1, adjusted for socio-demographic variables (i.e., age, school type, school grade, academic performance, parental highest educational attainment), screen time, physical activity, sleep, smoking status, alcohol use, and body mass index.

2 Model 2 is simultaneously adjusted for all independent variables.

## Supplementary materials

Supplementary Material 1. Predicted probabilities of mukbang viewing (≥ once/week) by personal, community, and societal level factors among Korean adolescents (n=36,990)

Supplementary Material 2. Prevalence ratios of mukbang viewing by personal, community, and societal level factors among Korean adolescents (n=36,990)

Supplementary Material 3. Predicted probabilities of mukbang viewing (≥ 3 times /week) by personal, community, and societal level factors among Korean adolescents (n=36,990)

Supplementary Material 4. Prevalence ratios of mukbang viewing by personal, community, and societal level factors among Korean adolescents (n=36,990)

Supplementary Material 5. Predicted probabilities of mukbang viewing (≥ 5 times /week) by personal, community, and societal level factors among Korean adolescents (n=36,990)

Supplementary Material 6. Prevalence ratios of mukbang viewing by personal, community, and societal level factors among Korean adolescents (n=36,990)
