# Supplementary material for: Personal, community, and societal factors associated with mukbang viewing among adolescents: findings from the Korea Youth Risk Behavior Survey
Source: Epidemiol Health. 2025 Sep 30;47:e2025055. doi: 10.4178/epih.e2025055 (PMC12869121; doi:10.4178/epih.e2025055)
Supplement: Supplementary Material 1. — Predicted probabilities of mukbang viewing (≥ once/week) by personal, community, and societal level factors among Korean adolescents (n=36,990) [file epih-47-e2025055-Supplementary-1.docx]

**Supplementary Material 1.** Predicted probabilities of *mukbang* viewing (≥ once/week) by personal, community, and societal level factors among Korean adolescents (n=36,990)

|  |  |  | ***Mukbang* viewing (≥ once/week)** | | | | | |
| --- | --- | --- | --- | --- | --- | --- | --- | --- |
|  |  |  | Predicted Probabilities (95% CI) | | | | | |
|  |  |  | Model 1 | | | Model 2 | | |
|  |  |  | **Total** | **Girls** | **Boys** | **Total** | **Girls** | **Boys** |
| Intrapersonal | Perceived health | Extremely healthy (N=7,624)  (Girls, n= 2,647, Boys, n=4,977) | 41.1  (39.9, 42.3) | 46.6  (44.7, 48.5) | 36.1  (34.7, 37.5) | 42.3  (41.1, 43.5) | 48.5  (46.5, 50.5) | 36.8  (35.4, 38.2) |
|  |  | Slightly healthy (N=16,141)  (Girls, n=7,958, Boys, n=8,183) | 42.0  (41.2, 42.8) | 47.3  (46.2, 48.4) | 37.2  (36.1, 38.3) | 42.1  (41.3, 42.9) | 47.6  (46.5, 48.7) | 37.2  (36.1, 38.3) |
|  |  | Normal (N=9,582)  (Girls, n=5,403, Boys, n=4,179) | 41.6  (40.6, 42.6) | 47.1  (45.7, 48.5) | 36.4  (34.9, 37.9) | 40.9  (39.9, 41.9) | 46.4  (45.0, 47.8) | 35.8  (34.3, 37.3) |
|  |  | Slightly unhealthy (N=3,439)  (Girls, n=1,871, Boys, n=1,568) | 41.3  (39.6, 43.0) | 47.1  (44.8, 49.4) | 35.4  (33.0, 37.8) | 39.5  (37.8, 41.2) | 45.0  (42.6, 47.4) | 34.3  (31.9, 36.8) |
|  |  | Extremely unhealthy (N=204)  (Girls, n=85, Boys, n=119) | 35.7  (29.0, 42.4) | 35.0  (24.9, 45.1) | 34.6  (33.7, 35.5) | 33.3  (26.8, 39.8) | 32.6  (22.7, 42.5) | 32.4  (24.0, 40.8) |
|  | Perceived weight | Extremely underweight (N=1,877)  (Girls, n=576, Boys, n=1,301) | 39.0  (36.5, 41.5) | 44.9  (44.5, 45.3) | 34.0  (31.0, 37.0) | 38.9  (36.4, 41.4) | 45.1  (44.6, 45.6) | 33.8  (30.8, 36.8) |
|  |  | Slightly underweight (N=8,242)  (Girls, n=3,517, Boys, n=4,725) | 38.9  (37.6, 40.2) | 45.5  (43.5, 47.5) | 33.0  (31.3, 34.7) | 38.9  (37.6, 40.2) | 45.6  (43.6, 47.6) | 32.9  (31.2, 34.6) |
|  |  | Normal weight (N=13,419)  (Girls, n=7,408, Boys, n=6,011) | 41.8  (40.9, 42.7) | 47.1  (45.9, 48.3) | 36.8  (35.5, 38.1) | 41.9  (41.0, 42.8) | 47.3  (46.1, 48.5) | 36.7  (35.4, 38.0) |
|  |  | Overweight (N=11,352)  (Girls, n=5,630, Boys, n=5,722) | 42.8  (41.7, 43.9) | 47.8  (46.2. 49.4) | 38.5  (36.9, 40.1) | 42.6  (41.4, 43.8) | 47.4  (45.8, 49.0) | 38.5  (36.9, 40.1) |
|  |  | Obesity (N=2,100)  (Girls, n=833, Boys, n=1,267) | 46.9  (44.1, 49.7) | 50.3  (46.1, 54.5) | 43.4  (39.7. 47.1) | 46.8  (44.0, 49.6) | 50.0  (45.8, 54.2) | 43.4  (39.7. 47.1) |
|  | Perceived stress | Low (N=6,230)  (Girls, n=2,324, Boys, n=3,906) | 37.0  (35.8, 38.2) | 42.4  (40.4, 44.4) | 32.4  (31.0, 33.8) | 37.8  (36.5, 39.1) | 44.2  (42.0. 46.4) | 32.3  (30.7, 33.9) |
|  |  | Moderate (N=15,615)  (Girls, n=7,235, Boys, n=8,380) | 41.3  (40.5, 42.1) | 45.9  (44.7, 47.1) | 37.0  (36.0, 38.0) | 41.6  (40.8, 42.4) | 46.7  (45.5, 47.9) | 37.1  (36.0, 38.2) |
|  |  | High (N=15,145)  (Girls, n=8,405, Boys, n=6,740) | 43.8  (43.0, 44.6) | 49.3  (48.2, 50.4) | 38.5  (37.3, 39.7) | 43.1  (42.2, 44.0) | 48.2  (47.0. 49.4) | 38.3  (37.0, 39.7) |
|  | Loneliness | Low (N=16,859)  (Girls, n=6,929, Boys, n=9,930) | 39.1  (38.3, 39.9) | 42.6  (41.4,43.8) | 35.5  (34.5, 36.5) | 40.0  (39.2, 40.8) | 43.4  (42.1, 44.7) | 36.4  (35.4, 37.4) |
|  |  | Moderate (N=13,751)  (Girls, n=7,228, Boys, n=6,523) | 43.4  (42.6, 44.2) | 49.3  (48.1, 50.5) | 37.9  (36.7, 39.1) | 43.0  (42.2, 43.8) | 49.0  (47.8, 50.2) | 37.2  (36.0, 38.4) |
|  |  | High (N=6,380)  (Girls, n=3,807, Boys, n=2,573) | 44.2  (42.9, 45.5) | 51.1  (49.5, 52.7) | 37.2  (35.3, 39.1) | 42.5  (41.1, 43.9) | 50.0  (48.2, 51.8) | 35.1  (33.0, 37.2) |
|  | Depression | No (N=26,547)  (Girls, n=12,050, Boys, n=14,497) | 40.1  (39.5, 40.7) | 45.1  (44.2, 46.0) | 35.5  (34.7, 36.3) | 40.5  (39.9, 41.1) | 45.8  (44.9. 46.7) | 35.6  (34.8, 36.4) |
|  |  | Yes (N=10,443)  (Girls, n=5,914, Boys, n=4,529) | 45.5  (44.5, 46.5) | 51.1  (49.8, 52.4) | 40.1  (38.6, 41.6) | 44.4  (43.3, 45.5) | 49.5  (48.1, 50.9) | 40.0  (38.4, 41.6) |
|  | Anxiety | Minimal (N=23,967)  (Girls, n=10,528, Boys, n=13,439) | 40.4  (39.8, 41.0) | 45.3  (44.3, 46.3) | 35.9  (35.1, 36.7) | 41.5  (40.8, 42.2) | 47.0  (45.9, 48.1) | 36.5  (35.6, 37.4) |
|  |  | Mild (N=8,723)  (Girls, n=4,814, Boys, n=3,909) | 44.2  (43.1, 45.3) | 49.8  (48.4, 51.2) | 38.9  (37.4, 40.4) | 42.6  (41.5, 43.7) | 48.1  (46.6, 49.6) | 37.5  (35.9, 39.1) |
|  |  | Moderate (N=3,047)  (Girls, n=1,829, Boys, n=1,218) | 43.5  (41.7, 45.3) | 50.0  (47.7, 52.3) | 37.2  (34.5, 39.9) | 40.3  (38.4, 42.2) | 46.1  (43.6, 48.6) | 34.8  (32.0, 37.6) |
|  |  | Severe (N=1,253)  (Girls, n=793, Boys, n=460) | 41.5  (38.7, 44.3) | 47.5  (44.0. 51.0) | 35.2  (30.5, 39.9) | 37.5  (34.7, 40.3) | 42.9  (39.2, 46.6) | 32.3  (27.9, 36.7) |
| Community | Nutrition education | No (N=19,707)  (Girls, n=9,677, Boys, n=10,030) | 40.1  (39.4, 40.8) | 45.5  (44.5, 46.5) | 35.1  (33.6, 36.6) | 40.0  (39.3, 40.7) | 45.5  (44.5, 46.5) | 35.1  (34.1, 36.1) |
|  |  | Yes (N=17,283)  (Girls, n=8,287, Boys, n=8,996) | 43.3  (42.5, 44.1) | 48.9  (47.8, 50.0) | 38.2  (37.2, 39.2) | 43.3  (42.5, 44.1) | 48.9  (47.8, 50.0) | 38.1  (37.0, 39.0) |
|  | Living arrangement | Family members (N=35,515)  (Girls, n=17,332, Boys, n=18,183) | 41.5  (41.0, 42.0) | 47.1  (46.4, 47.8) | 36.3  (35.6, 37.0) | 41.5  (41.0, 42.0) | 47.1  (46.4, 47.8) | 36.3  (29.2, 43.4) |
|  |  | Relatives (N=159)  (Girls, n=62, Boys, n=97) | 39.2  (31.3, 47.1) | 42.0  (29.5, 54.5) | 35.8  (26.0, 45.6) | 39.3  (31.4, 47.2) | 41.5  (28.9, 54.1) | 36.1  (26.2, 46.0) |
|  |  | Off campus (N=215)  (Girls, n=84, Boys, n=131) | 50.8  (43.8, 57.8) | 50.4  (39.4, 61.4) | 49.7  (40.7, 58.7) | 50.1  (43.1, 57.1) | 49.4  (38.4, 60.4) | 49.4  (40.4, 58.4) |
|  |  | On campus (N=1,025)  (Girls, n=451, Boys, n=574) | 42.8  (39.7, 45.9) | 45.4  (40.7, 50.1) | 40.4  (36.3, 44.5) | 42.4  (42.1, 42.7) | 45.0  (40.2, 49.8) | 40.1  (36.0, 44.2) |
| Societal | Socioeconomic status | High (N=4,349)  (Girls, n=1,818, Boys, n=2,531) | 41.7  (37.7, 45.7) | 44.2  (41.8, 46.6) | 36.5  (34.5, 38.5) | 40.5  (36.5, 44.5) | 44.4  (42.0, 46.8) | 36.4  (34.4, 38.4) |
|  |  | Medium (N=32,131)  (Girls, n=15,989, Boys, n=16,142) | 41.7  (41.2, 42.2) | 47.4  (46.6, 48.2) | 36.6  (35.8, 37.4) | 41.7  (41.2, 42.2) | 47.3  (46.6, 48.2) | 36.6  (35.8, 37.4) |
|  |  | Low (N=601)  (Girls, n=248, Boys, n=353) | 40.4  (38.9, 41.9) | 49.3  (42.9, 55.7) | 35.2  (30.2, 40.2) | 40.4  (38.9, 41.9) | 57.7  (51.3. 64.1) | 34.0  (29.0, 39.0) |

Note: In model 1, each value represents predicted probability (95% CI) adjusted for sociodemographic variables (i.e., age, school type, school grade, academic performance, parental maximum educational attainment), screen-time, physical activity, sleep, smoking status, alcohol use, and BMI. For each variable, the first category listed serves as the referent group

Model 2 simultaneously adjusts for all independent variables
