# Supplementary material for: Personal, community, and societal factors associated with mukbang viewing among adolescents: findings from the Korea Youth Risk Behavior Survey
Source: Epidemiol Health. 2025 Sep 30;47:e2025055. doi: 10.4178/epih.e2025055 (PMC12869121; doi:10.4178/epih.e2025055)
Supplement: Supplementary Material 2. — Prevalence ratios of mukbang viewing by personal, community, and societal level factors among Korean adolescents (n=36,990) [file epih-47-e2025055-Supplementary-2.docx]

**Supplementary Material 2.** Prevalence ratios of *mukbang* viewing by personal, community, and societal level factors among Korean adolescents (n=36,990)

|  |  |  | ***Mukbang* viewing (≥ once/week)** | | | | | |
| --- | --- | --- | --- | --- | --- | --- | --- | --- |
|  |  |  | Prevalence Ratios (95% CI) | | | | | |
|  |  |  | Model 1 | | | Model 2 | | |
|  |  |  | **Total** | **Girls** | **Boys** | **Total** | **Girls** | **Boys** |
| Intrapersonal | Perceived health | Extremely healthy (N=7,624)  (Girls, n= 2,647, Boys, n=4,977) | **1.00 (ref)** | **1.00 (ref)** | **1.00 (ref)** | **1.00 (ref)** | **1.00 (ref)** | **1.00 (ref)** |
|  |  | Slightly healthy (N=16,141)  (Girls, n=7,958, Boys, n=8,183) | 1.02  (0.98-1.07) | 1.01  (0.95-1.08) | 1.03  (0.97-1.09) | 1.00  (0.95-1.04) | 0.98  (0.92-1.05) | 1.01  (0.95-1.07) |
|  |  | Normal (N=9,582)  (Girls, n=5,403, Boys, n=4,179) | 1.01  (0.96-1.06) | 1.01  (0.94-1.08) | 1.01  (0.94-1.08) | 0.97  (0.92-1.02) | 0.96  (0.89-1.03) | 0.98  (0.91-1.05) |
|  |  | Slightly unhealthy (N=3,439)  (Girls, n=1,871, Boys, n=1,568) | 1.00  (0.94-1.07) | 1.01  (0.92-1.10) | 0.98  (0.89-1.08) | 0.94  (0.88-1.00) | 0.93  (0.85-1.01) | 0.93  (0.84-1.03) |
|  |  | Extremely unhealthy (N=204)  (Girls, n=85, Boys, n=119) | 0.87  (0.69-1.10) | 0.77  (0.54-1.09) | 0.96  (0.70-1.30) | 0.80  (0.63-1.01) | 0.70  (0.49-0.99) | 0.89  (0.65-1.21) |
|  | Perceived weight | Extremely underweight (N=1,877)  (Girls, n=576, Boys, n=1,301) | **1.00 (ref)** | **1.00 (ref)** | **1.00 (ref)** | **1.00 (ref)** | **1.00 (ref)** | **1.00 (ref)** |
|  |  | Slightly underweight (N=8,242)  (Boys, n=4,725, Girls, n=3,517) | 1.00  (0.92-1.09) | 1.01  (0.89-1.15) | 0.98  (0.88-1.09) | 1.00  (0.92-1.09) | 1.01  (0.89-1.15) | 0.98  (0.88-1.09) |
|  |  | Normal weight (N=13,419)  (Girls, n=7,408, Boys, n=6,011) | 1.07  (0.99-1.17) | 1.05  (0.92-1.20) | 1.08  (0.97-1.21) | 1.08  (0.99-1.17) | 1.05  (0.92-1.20) | 1.09  (0.97-1.22) |
|  |  | Overweight (N=11,352)  (Girls, n=5,630, Boys, n=5,722) | 1.09  (0.99-1.20) | 1.06  (0.92-1.23) | 1.13  (0.99-1.29) | 1.09  (0.99-1.20) | 1.05  (0.91-1.22) | 1.14  (1.00-1.30) |
|  |  | Obesity (N=2,100)  (Girls, n=833, Boys, n=1,267) | 1.19  (1.05-1.36) | 1.12  (0.92-1.35) | 1.27  (1.06-1.52) | 1.19  (1.04-1.36) | 1.10  (0.91-1.34) | 1.28  (1.07-1.53) |
|  | Perceived stress | Low (N=6,230)  (Girls, n=2,324, Boys, n=3,906) | **1.00 (ref)** | **1.00 (ref)** | **1.00 (ref)** | **1.00 (ref)** | **1.00 (ref)** | **1.00 (ref)** |
|  |  | Moderate (N=15,615)  (Girls, n=7,235, Boys, n=8,380) | 1.11  (1.06-1.17) | 1.08  (1.01-1.16) | 1.14  (1.07-1.22) | 1.10  (1.05-1.16) | 1.06  (0.98-1.14) | 1.14  (1.04-1.22) |
|  |  | High (N=15,145)  (Girls, n=8,405, Boys, n=6,740) | 1.18  (1.12-1.24) | 1.16  (1.08-1.24) | 1.19  (1.11-1.27) | 1.14  (1.08-1.20) | 1.09  (1.01-1.18) | 1.18  (1.09-1.27) |
|  | Loneliness | Low (N=16,859)  (Girls, n=6,929, Boys, n=9,930) | **1.00 (ref)** | **1.00 (ref)** | **1.00 (ref)** | **1.00 (ref)** | **1.00 (ref)** | **1.00 (ref)** |
|  |  | Moderate (N=13,751)  (Girls, n=7,228, Boys, n=6,523) | 1.11  (1.07-1.15) | 1.15  (1.10-1.21)_ | 1.07  (1.01-1.21) | 1.07  (1.03-1.11) | 1.13  (1.07-1.19) | 1.02  (0.97-1.08) |
|  |  | High (N=6,380)  (Girls, n=3,807, Boys, n=2,573) | 1.12  (1.08-1.18) | 1.19  (1.13-1.26) | 1.05  (0.98-1.13) | 1.06  (1.01-1.12) | 1.14  (1.07-1.23) | 0.97  (0.89-1.05) |
|  | Depression | No (N=26,547)  (Girls, n=12,050, Boys, n=14,497) | **1.00 (ref)** | **1.00 (ref)** | **1.00 (ref)** | **1.00 (ref)** | **1.00 (ref)** | **1.00 (ref)** |
|  |  | Yes (N=10,443)  (Boys, n=4,529, Girls, n=5,914) | 1.13  (1.09-1.17) | 1.13  (1.08-1.18) | 1.13  (1.07-1.19) | 1.09  (1.05-1.13) | 1.08  (1.02-1.14) | 1.10  (1.05-1.18) |
|  | Anxiety | Minimal (N=23,967)  (Girls, n=10,528, Boys, n=13,439) | **1.00 (ref)** | **1.00 (ref)** | **1.00 (ref)** | **1.00 (ref)** | **1.00 (ref)** | **1.00 (ref)** |
|  |  | Mild (N=8,723)  (Girls, n=4,814, Boys, n=3,909) | 1.09  (1.05-1.13) | 1.10  (1.05-1.15) | 1.08  (1.02-1.15) | 1.02  (0.98-1.07) | 1.05  (0.97-1.08) | 1.03  (0.96-1.09) |
|  |  | Moderate (N=3,047)  (Girls, n=1,829, Boys, n=1,218) | 1.07  (1.01-1.13) | 1.09  (1.02-1.17) | 1.04  (0.95-1.14) | 0.97  (0.91-1.03) | 0.98  (0.91-1.06) | 0.96  (0.86-1.06) |
|  |  | Severe (N=1,253)  (Girls, n=793, Boys, n=460) | 1.02  (0.94-1.11) | 1.05  (0.94-1.16) | 0.98  (0.84-1.15) | 0.91  (0.83-1.00) | 0.92  (0.82-1.03) | 0.89  (0.76-1.05) |
| Community | Nutrition education | No (N=19,707)  (Girls, n=9,677, Boys, n=10,030) | **1.00 (ref)** | **1.00 (ref)** | **1.00 (ref)** | **1.00 (ref)** | **1.00 (ref)** | **1.00 (ref)** |
|  |  | Yes (N=17,283)  (Boys, n=8,996, Girls, n=8,287) | 1.08  (1.04-1.11) | 1.07  (1.03-1.12) | 1.09  (1.03-1.14) | 1.08  (1.04-1.11) | 1.07  (1.03-1.12) | 1.08  (1.03-1.14) |
|  | Living arrangement | Family members (N=35,515)  (Girls, n=17,332, Boys, n=18,183) | **1.00 (ref)** | **1.00 (ref)** | **1.00 (ref)** | **1.00 (ref)** | **1.00 (ref)** | **1.00 (ref)** |
|  |  | Relatives (N=159)  (Girls, n=62, Boys, n=97) | 0.93  (0.72-1.20) | 0.87  (0.59-1.28) | 0.97  (0.69-1.37) | 0.93  (0.72-1.20) | 0.86  (0.59-1.27) | 0.98  (0.70-1.38) |
|  |  | Off campus (N=215)  (Girls, n=84, Boys, n=131) | 1.20  (0.99-1.45) | 1.05  (0.78-1.42) | 1.32  (1.04-1.70) | 1.18  (0.98-1.43) | 1.04  (0.77-1.40) | 1.31  (1.03-1.68) |
|  |  | On campus (N=1,025)  (Girls, n=451, Boys, n=574) | 1.03  (0.93-1.13) | 0.97  (0.84-1.16) | 1.10  (0.96-1.26) | 1.02  (0.93-1.12) | 0.96  (0.83-1.11) | 1.09  (0.96-1.25) |
| Societal | Socioeconomic status | High (N=4,349)  (Girls, n=1,818, Boys, n=2,531) | **1.00 (ref)** | **1.00 (ref)** | **1.00 (ref)** | **1.00 (ref)** | **1.00 (ref)** | **1.00 (ref)** |
|  |  | Medium (N=32,131)  (Girls, n=15,989, Boys, n=16,142) | 1.03  (0.98-1.08) | 1.07  (0.99-1.15) | 1.10  (0.93-1.07) | 1.03  (0.97-1.08) | 1.06  (0.98-1.15) | 1.00  (0.93-1.07) |
|  |  | Low (N=601)  (Girls, n=248, Boys, n=353) | 1.01  (0.89-1.15) | 1.08  (0.90-1.31) | 0.95  (0.79-1.13) | 0.98  (0.86-1.12) | 1.06  (0.87-1.28) | 0.92  (0.76-1.10) |

Note: In model 1, each value represents prevalence ratio (95% CI) adjusted for sociodemographic variables (i.e., age, school type, school grade, academic performance, parental maximum educational attainment), screen-time, physical activity, sleep, smoking status, alcohol use, and BMI. For each variable, the first category listed serves as the referent group

Model 2 simultaneously adjusts for all independent variables
