# Supplementary material for: Personal, community, and societal factors associated with mukbang viewing among adolescents: findings from the Korea Youth Risk Behavior Survey
Source: Epidemiol Health. 2025 Sep 30;47:e2025055. doi: 10.4178/epih.e2025055 (PMC12869121; doi:10.4178/epih.e2025055)
Supplement: Supplementary Material 3. — Predicted probabilities of mukbang viewing (≥ 3 times /week) by personal, community, and societal level factors among Korean adolescents (n=36,990) [file epih-47-e2025055-Supplementary-3.docx]

**Supplementary Material 3.** Predicted probabilities of *mukbang* viewing (≥ 3 times /week) by personal, community, and societal level factors among Korean adolescents (n=36,990)

|  |  |  | ***Mukbang* viewing (≥ 3 times/week)** | | | | | |
| --- | --- | --- | --- | --- | --- | --- | --- | --- |
|  |  |  | Predicted Probabilities (95% CI) | | | | | |
|  |  |  | Model 1 | | | Model 2 | | |
|  |  |  | **Total** | **Girls** | **Boys** | **Total** | **Girls** | **Boys** |
| Intrapersonal | Perceived health | Extremely healthy (N=7,624)  (Girls, n= 2,647, Boys, n=4,977) | 23.5  (22.5, 24.5) | 27.3  (25.6, 29.0) | 20.2  (19.1, 21.3) | 24.5  (23.4, 25.6) | 28.8  (27.0, 30.6) | 20.1  (18.8, 21.4) |
|  |  | Slightly healthy (N=16,141)  (Girls, n=7,958, Boys, n=8,183) | 23.3  (22.7, 23.9) | 27.5  (26.5, 28.5) | 19.8  (18.9, 20.7) | 23.3  (22.6, 24.0) | 27.7  (26.7, 28.7) | 19.7  (18.8, 20.6) |
|  |  | Normal (N=9,582)  (Girls, n=5,403, Boys, n=4,179) | 23.3  (22.4, 24.2) | 27.2  (26.0, 28.4) | 19.9  (18.7, 21.1) | 22.6  (21.7, 23.5) | 26.5  (25.3, 27.7) | 19.3  (18.1, 20.5) |
|  |  | Slightly unhealthy (N=3,439)  (Girls, n=1,871, Boys, n=1,568) | 25.2  (23.7, 26.7) | 29.4  (27.3, 31.5) | 21.1  (19.1, 23.1) | 23.4  (22.0, 24.8) | 27.3  (25.2, 29.4) | 19.8  (17.8. 21.8) |
|  |  | Extremely unhealthy (N=204)  (Girls, n=85, Boys, n=119) | 27.4  (21.2, 33.6) | 27.8  (18.4, 37.2) | 25.7  (17.8, 33.6) | 24.7  (18.9, 30.5) | 25.1  (16.2, 34.0) | 23.2  (15.8, 30.6) |
|  | Perceived weight | Extremely underweight (N=1,877)  (Girls, n=576, Boys, n=1,301) | 21.9  (19.8, 24.0) | 26.6  (22.7. 30.5) | 18.3  (15.9, 20.7) | 21.6  (19.5, 23.7) | 26.5  (22.6, 30.4) | 17.9  (15.5. 20.3) |
|  |  | Slightly underweight (N=8,242)  (Girls, n=3,517, Boys, n=4,725) | 21.1  (20.0, 22.2) | 25.4  (23.6, 27.2) | 17.3  (16.0, 18.6) | 21.0  (19.9, 22.1) | 25.4  (23.7, 27.1) | 17.2  (15.9, 18.5) |
|  |  | Normal weight (N=13,419)  (Girls, n=7,408, Boys, n=6,011) | 23.6  (22.9, 24.3) | 28.0  (26.9, 29.1) | 19.4  (18.4, 20.4) | 23.6  (22.9, 24.3) | 28.2  (27.1, 29.3) | 19.4  (18.4, 20.4) |
|  |  | Overweight (N=11,352)  (Girls, n=5,630, Boys, n=5,722) | 24.8  (23.8, 25.8) | 28.2  (26.7, 29.7) | 22.3  (20.9, 23.7) | 24.5  (23.5, 25.5) | 27.7  (26.3, 29.1) | 22.1  (20.7, 23.5) |
|  |  | Obesity (N=2,100)  (Girls, n=833, Boys, n=1,267) | 27.9  (25.4, 30.4) | 29.3  (25.5. 33.1) | 26.4  (23.0, 29.7 | 27.5  (25.0, 30.0) | 28.6  (24.9. 32.3) | 26.3 (22.9,29.7) |
|  | Perceived stress | Low (N=6,230)  (Girls, n=2,324, Boys, n=3,906) | 19.8  (18.8, 20.8) | 24.2  (22.3, 26.1) | 16.4  (15.2, 17.6) | 20.5  (19.4, 21.6) | 25.9  (23.9, 27.9) | 16.4  (15.2, 17.6) |
|  |  | Moderate (n=15,615)  (Girls, n=7,235, Boys, n=8,380) | 22.8  (22.1, 23.5) | 26.1  (25.1, 27.1) | 19.9  (19.0, 20.8) | 23.0  (22.3, 23.7) | 26.7  (25.6, 27.8) | 19.9  (19.0. 20.8) |
|  |  | High (n=15,145)  (Girls, n=8,405, Boys, n=6,740) | 25.9  (25.2, 26.6) | 29.8  (28.8, 30.8) | 22.4  (21.4, 23.4) | 25.1  (24.3, 25.9) | 28.5  (27.4, 29.6) | 22.2  (21.1, 23.3) |
|  | Loneliness | Low (N=16,859)  (Girls, n=6,929, Boys, n=9,930) | 21.6  (21.0, 22.2) | 24.2  (23.2, 25.2) | 19.1  (18.3, 19.9) | 22.4  (21.7, 23.1) | 25.1  (24.0, 26.2) | 19.9  (19.1, 20.7) |
|  |  | Moderate (N=13,751)  (Girls, n=7,228, Boys, n=6,523) | 24.4  (23.7, 25.1) | 28.6  (27.5, 29.7) | 20.7  (19.7, 21.7) | 23.9  (23.2, 24.6) | 28.3  (27.2. 29.4) | 20.0  (19.0, 21.0) |
|  |  | High (N=6,380)  (Girls, n=3,807, Boys, n=2,573) | 26.8  (25.7, 27.9) | 31.8  (30.3, 33.3) | 21.9  (20.3, 23.5) | 24.9  (23.7, 26.1) | 30.2  (28.5, 31.9) | 19.9  (18.2, 21.6) |
|  | Depression | No (N=26,547)  (Girls, n=12,050, Boys, n=14,497) | 22.2  (21.7, 22.7) | 25.8  (25.0, 26.6) | 19.2  (18.6, 19.8) | 22.6  (22.1, 23.1) | 26.4  (25.6. 27.2) | 19.3  (18.6, 20.0) |
|  |  | Yes (N=10,443)  (Girls, n=5,914, Boys, n=4,529) | 26.9  (26.0, 27.8) | 31.3  (30.1, 32.5) | 22.9  (21.7, 24.1) | 25.6  (24.7, 26.5) | 29.7  (28.4, 31.0) | 21.9  (20.6, 23.2) |
|  | Anxiety | Minimal (N=23,967)  (Girls, n=10,528, Boys, n=13,439) | 22.3  (21.8, 22.8) | 25.7  (25.0, 26.5) | 19.3  (18.6, 20.0) | 23.2  (22.6, 23.8) | 26.9  (26.0, 27.8) | 19.9  (19.2, 20.6) |
|  |  | Mild (n=8,723)  (Girls, n=4,814, Boys, n=3,909) | 26.1  (25.2, 27.0) | 30.4  (29.1, 31.7) | 22.2  (20.9, 22.4) | 24.5  (23.5, 25.5) | 28.9  (27.6, 30.2) | 20.6  (19.3, 21.9) |
|  |  | Moderate (n=3,047)  (Girls, n=1,829, Boys, n=1,218) | 25.9  (24.4, 27.4) | 30.4  (28.3, 32.5) | 21.8  (19.5, 24.1) | 23.1  (21.6, 24.6) | 27.4  (25.2, 29.6) | 19.2  (16.9. 21.5) |
|  |  | Severe (n=1,253)  Girls, n=793, Boys, n=460) | 23.8  (21.5, 26.1) | 28.6  (25.5, 31.7) | 18.7  (15.2, 22.2) | 20.5  (18.3, 22.7) | 24.8  (21.8, 27.8) | 16.1  (12.8, 19.4) |
| Community | Nutrition education | No (N=19,707)  (Girls, n=9,677, Boys, n=10,030) | 22.9  (22.3, 23.5) | 26.6  (25.7, 27.5) | 19.7  (18.9, 20.5) | 22.7  (22.1, 23.3) | 26.5  (25.6, 27.4) | 19.5  (18.7, 20.3) |
|  |  | Yes (N=17,283)  (Girls, n=8,287, Boys, n=8,996) | 24.3  (23.6, 25.0) | 28.7  (27.7, 39.7) | 20.5  (19.6, 21.4) | 24.2  (23.5, 24.9) | 28.6  (27.6, 39.6) | 20.3  (19.4, 21.2) |
|  | Living arrangement | Family members (N=35,515)  (Girls, n=17,332, Boys, n=18,183) | 23.3  (22.8, 23.8) | 27.5  (26.8, 28.2) | 19.9  (19.3, 20.5) | 23.2  (22.7, 23.7) | 27.4  (26.7, 28.1) | 19.7  (19.1, 20.3) |
|  |  | Relatives (N=159)  (Girls, n=62, Boys, n=97) | 24.8  (17.9, 31.7) | 23.5  (12.9, 34.1) | 24.3  (15.6, 33.0) | 24.7  (17.8, 31.6) | 23.0  (12.5, 33.5) | 24.6  (15.9, 33.3) |
|  |  | Off campus (N=215)  (Girls, n=84, Boys, n=131) | 30.3  (24.0, 36.6) | 30.3  (20.3, 40.3) | 29.5  (21.5. 37.5) | 29.6  (23.4, 35.8) | 29.4  (19.6, 39.2) | 29.2  (21.2, 37.2) |
|  |  | On campus (N=1,025)  (Girls, n=451, Boys, n=574) | 26.6  (23.8, 29.4) | 31.1  (26.7, 35.5) | 23.2  (19.7, 26.7) | 26.2  (23.4, 29.0) | 30.6  (26.2, 35.0) | 22.9  (19.4, 26.4) |
| Societal | Socioeconomic status | High (N=4,349)  (Girls, n=1,818, Boys, n=2,531) | 25.2  (21.8, 28.6) | 25.9  (23.8, 28.0) | 21.0  (19.3, 22.7) | 23.7  (20.4, 27.0) | 26.0  (23.9, 28.1) | 20.9  (19.2, 22.6) |
|  |  | Medium (N=32,131)  (Girls, n=15,989, Boys, n=16,142) | 23.5  (23.0, 24.0) | 27.8  (27.1, 28.5) | 19.9  (19.3, 20.5) | 23.3  (22.8, 23.8) | 27.6  (26.9, 28.3) | 19.7  (19.1, 20.3) |
|  |  | Low (N=601)  (Girls, n=248, Boys, n=353) | 23.4  (22.1, 24.7) | 27.4  (22.0, 32.8) | 22.8  (18.5, 27.1) | 23.5  (22.2, 24.8) | 25.9  (20.6, 31.2) | 21.4  (17.3, 25.5) |

Note: In model 1, each value represents predicted probability (95% CI) adjusted for sociodemographic variables (i.e., age, school type, school grade, academic performance, parental maximum educational attainment), screen-time, physical activity, sleep, smoking status, alcohol use, and BMI. For each variable, the first category listed serves as the referent group

Model 2 simultaneously adjusts for all independent variables
