# Supplementary material for: Personal, community, and societal factors associated with mukbang viewing among adolescents: findings from the Korea Youth Risk Behavior Survey
Source: Epidemiol Health. 2025 Sep 30;47:e2025055. doi: 10.4178/epih.e2025055 (PMC12869121; doi:10.4178/epih.e2025055)
Supplement: Supplementary Material 4. — Prevalence ratios of mukbang viewing by personal, community, and societal level factors among Korean adolescents (n=36,990) [file epih-47-e2025055-Supplementary-4.docx]

**Supplementary Material 4.** Prevalence ratios of *mukbang* viewing by personal, community, and societal level factors among Korean adolescents (n=36,990)

|  |  |  | **M*ukbang* viewing (≥ 3 times/week)** | | | | | |
| --- | --- | --- | --- | --- | --- | --- | --- | --- |
|  |  |  | Prevalence Ratios (95% CI) | | | | | |
|  |  |  | Model 1 | | | Model 2 | | |
|  |  |  | **Total** | **Girls** | **Boys** | **Total** | **Girls** | **Boys** |
| Intrapersonal | Perceived health | Extremely healthy (N=7,624)  (Girls, n= 2,647, Boys, n=4,977) | **1.00 (ref)** | **1.00 (ref)** | **1.00 (ref)** | **1.00 (ref)** | **1.00 (ref)** | **1.00 (ref)** |
|  |  | Slightly healthy (N=16,141)  (Girls, n=7,958, Boys, n=8,183) | 0.99  (0.93-1.05) | 1.00  (0.92-1.09) | 0.98  (0.90-1.06) | 0.95  (0.90-1.01) | 0.96  (0.88-1.05) | 0.95  (0.88-1.03) |
|  |  | Normal (N=9,582)  (Girls, n=5,403, Boys, n=4,179) | 0.99  (0.93-1.05) | 0.99  (0.91-1.09) | 0.98  (0.90-1.08) | 0.93  (0.87-0.99) | 0.92  (0.84-1.01) | 0.93  (0.84-1.02) |
|  |  | Slightly unhealthy (N=3,439)  (Girls, n=1,871, Boys, n=1,568) | 1.06  (0.98-1.15) | 1.07  (0.96-1.19) | 1.04  (0.92-1.18) | 0.95  (0.87-1.04) | 0.94  (0.84-1.06) | 0.95  (0.83-1.08) |
|  |  | Extremely unhealthy (N=204)  (Girls, n=85, Boys, n=119) | 1.15  (0.89-1.50) | 1.01  (0.68-1.50) | 1.25  (0.88-1.79) | 1.00  (0.77-1.30) | 0.87  (0.59-1.30) | 1.09  (0.76-1.56) |
|  | Perceived weight | Extremely underweight (N=1,877)  (Girls, n=576, Boys, n=1,301) | **1.00 (ref)** | **1.00 (ref)** | **1.00 (ref)** | **1.00 (ref)** | **1.00 (ref)** | **1.00 (ref)** |
|  |  | Slightly underweight (N=8,242)  (Girls, n=3,517, Boys, n=4,725) | 0.97  (0.87-1.08) | 0.96  (0.81-1.13) | 0.96  (0.83-1.10) | 0.97  (0.87-1.09) | 0.96  (0.81-1.14) | 0.97  (0.84-1.12) |
|  |  | Normal weight (N=13,419)  (Girls, n=7,408, Boys, n=6,011) | 1.08  (0.96-1.20) | 1.05  (0.89-1.25) | 1.07  (0.92-1.24) | 1.09  (0.98-1.22) | 1.06  (0.90-1.26) | 1.08  (0.93-1.26_ |
|  |  | Overweight (N=11,352)  (Girls, n=5,630, Boys, n=5,722) | 1.13  (0.99-1.28) | 1.06  (0.88-1.28) | 1.22  (1.02-1.46) | 1.13  (0.99-1.28) | 1.05  (0.87-1.26) | 1.23  (1.03-1.47) |
|  |  | Obesity (N=2,100)  (Girls, n=833, Boys, n=1,267) | 1.26  (1.06-1.49) | 1.10  (0.85-1.40) | 1.44  (1.13-1.82) | 1.25  (1.06-1.48) | 1.08  (0.84-1.38) | 1.45  (1.15-1.84) |
|  | Perceived stress | Low (N=6,230)  (Girls, n=2,324, Boys, n=3,906) | **1.00 (ref)** | **1.00 (ref)** | **1.00 (ref)** | **1.00 (ref)** | **1.00 (ref)** | **1.00 (ref)** |
|  |  | Moderate (N=15,615)  (Girls, n=7,235, Boys, n=8,380) | 1.15  (1.07-1.23) | 1.08  (0.98-1.18) | 1.21  (1.11-1.33) | 1.12  (1.05-1.20) | 1.04  (0.94-1.14) | 1.21  (1.10-1.33) |
|  |  | High (N=15,145)  (Girls, n=8,405, Boys, n=6,740) | 1.30  (1.22-1.38) | 1.22  (1.11-1.34) | 1.36  (1.24-1.50) | 1.22  (1.13-1.31) | 1.10  (0.99-1.22) | 1.34  (1.21-1.49) |
|  | Loneliness | Low (N=16,859)  (Girls, n=6,929, Boys, n=9,930) | **1.00 (ref)** | **1.00 (ref)** | **1.00 (ref)** | **1.00 (ref)** | **1.00 (ref)** | **1.00 (ref)** |
|  |  | Moderate (N=13,751)  (Girls, n=7,228, Boys, n=6,523) | 1.13  (1.08-1.18) | 1.22  (1.14-1.30) | 1.12  (1.04-1.20) | 1.07  (1.02-1.12) | 1.13  (1.05-1.21) | 1.01  (0.94-1.09) |
|  |  | High (N=6,380)  (Girls, n=3,807, Boys, n=2,573) | 1.23  (1.16-1.30) | 1.40  (1.30-1.51) | 1.23  (1.12-1.34) | 1.10  (1.03-1.18) | 1.20  (1.09-1.31) | 1.00  (0.90-1.12) |
|  | Depression | No (N=26,547)  (Girls, n=12,050, Boys, n=14,497) | **1.00 (ref)** | **1.00 (ref)** | **1.00 (ref)** | **1.00 (ref)** | **1.00 (ref)** | **1.00 (ref)** |
|  |  | Yes (N=10,443)  (Girls, n=5,914, Boys, n=4,529) | 1.20  (1.15-1.26) | 1.21  (1.14-1.28) | 1.19  (1.11-1.28) | 1.13  (1.07-1.19) | 1.13  (1.05-1.20) | 1.13  (1.04-1.22) |
|  | Anxiety | Minimal (N=23,967)  (Girls, n=10,528, Boys, n=13,439) | **1.00 (ref)** | **1.00 (ref)** | **1.00 (ref)** | **1.00 (ref)** | **1.00 (ref)** | **1.00 (ref)** |
|  |  | Mild (N=8,723)  (Girls, n=4,814, Boys, n=3,909) | 1.17  (1.11-1.22) | 1.18  (1.11-1.26) | 1.14  (1.06-1.24) | 1.06  (1.01-1.11) | 1.07  (1.00-1.15) | 1.04  (0.95-1.13) |
|  |  | Moderate (N=3,047)  (Girls, n=1,829, Boys, n=1,218) | 1.15  (1.07-1.24) | 1.18  (1.08-1.29) | 1.13  (1.00-1.28) | 0.99  (0.92-1.08) | 1.01  (0.92-1.12) | 0.97  (0.85-1.11) |
|  |  | Severe (N=1,253)  (Girls, n=793, Boys, n=460) | 1.06  (0.95-1.19) | 1.11  (0.97-1.26) | 0.97  (0.79-1.19) | 0.89  (0.79-1.01) | 0.93  (0.80-1.07) | 0.82 (0.661.02) |
| Community | Nutrition education | No (N=19,707)  (Girls, n=9,677, Boys, n=10,030) | **1.00 (ref)** | **1.00 (ref)** | **1.00 (ref)** | **1.00 (ref)** | **1.00 (ref)** | **1.00 (ref)** |
|  |  | Yes (N=17,283)  (Girls, n=8,287, Boys, n=8,996) | 1.06  (1.01-1.11) | 1.08  (1.02-1.14) | 1.04  (0.97-1.11) | 1.06  (1.01-1.11) | 1.08  (1.02-1.14) | 1.04  (0.97-1.11) |
|  | Living arrangement | Family members (N=35,515)  (Girls, n=17,332, Boys, n=18,183) | **1.00 (ref)** | **1.00 (ref)** | **1.00 (ref)** | **1.00 (ref)** | **1.00 (ref)** | **1.00 (ref)** |
|  |  | Relatives (N=159)  (Girls, n=62, Boys, n=97) | 1.03  (0.75-1.41) | 0.83  (0.50-1.38) | 1.19  (0.79-1.78) | 1.03  (0.75-1.41) | 0.82  (0.19-1.37) | 1.20  (0.80-1.80) |
|  |  | Off campus (N=215)  (Girls, n=84, Boys, n=131) | 1.26  (0.99-1.60) | 1.08  (0.74-1.58) | 1.44  (1.05-1.96) | 1.23  (0.97-1.57) | 1.05  (0.72-1.54) | 1.42  (1.04-1.94) |
|  |  | On campus (N=1,025)  (Girls, n=451, Boys, n=574) | 1.13  (1.00-1.27) | 1.13  (0.95-1.34) | 1.15  (0.97-1.37) | 1.12  (0.99-1.26) | 1.12  (0.94-1.32) | 1.15  (0.96-1.36) |
| Societal | Socioeconomic status | High (N=4,349)  (Girls, n=1,818, Boys, n=2,531) | **1.00 (ref)** | **1.00 (ref)** | **1.00 (ref)** | **1.00 (ref)** | **1.00 (ref)** | **1.00 (ref)** |
|  |  | Medium (N=32,131)  (Girls, n=15,989, Boys, n=16,142) | 0.99  (0.93-1.06) | 1.06  (0.96-1.18) | 0.93  (0.85-1.03) | 0.99  (0.92-1.06) | 1.05  (0.95-1.16) | 0.93  (0.85-1.03) |
|  |  | Low (N=601)  (Girls, n=248, Boys, n=353) | 1.03  (0.87-1.22) | 1.02  (0.80-1.31) | 1.04  (0.83-1.30) | 0.97  (0.82-1.15) | 0.97  (0.76-1.24) | 0.98  (0.78-1.22) |

Note: In model 1, each value represents prevalence ratio (95% CI) adjusted for sociodemographic variables (i.e., age, school type, school grade, academic performance, parental maximum educational attainment), screen-time, physical activity, sleep, smoking status, alcohol use, and BMI. For each variable, the first category listed serves as the referent group

Model 2 simultaneously adjusts for all independent variables
