# Supplementary material for: Personal, community, and societal factors associated with mukbang viewing among adolescents: findings from the Korea Youth Risk Behavior Survey
Source: Epidemiol Health. 2025 Sep 30;47:e2025055. doi: 10.4178/epih.e2025055 (PMC12869121; doi:10.4178/epih.e2025055)
Supplement: Supplementary Material 5. — Predicted probabilities of mukbang viewing (≥ 5 times /week) by personal, community, and societal level factors among Korean adolescents (n=36,990) [file epih-47-e2025055-Supplementary-5.docx]

**Supplementary Material 5.** Predicted probabilities of *mukbang* viewing (≥ 5 times /week) by personal, community, and societal level factors among Korean adolescents (n=36,990)

|  |  |  | ***Mukbang* viewing (≥ 5 times /week)** | | | | | |
| --- | --- | --- | --- | --- | --- | --- | --- | --- |
|  |  |  | Predicted Probabilities (95% CI) | | | | | |
|  |  |  | Model 1 | | | Model 2 | | |
|  |  |  | **Total** | **Girls** | **Boys** | **Total** | **Girls** | **Boys** |
| Intrapersonal | Perceived health | Extremely healthy (N=7,624)  (Girls, n= 2,647, Boys, n=4,977) | 12.2  (11.4, 13.0) | 14.4  (13.0, 15.8) | 10.5  (9.6, 11.4) | 12.9  (12.1, 13.7) | 15.3  (13.8, 16.8) | 10.9 (10.0,11.8) |
|  |  | Slightly healthy (N=16,141)  (Girls, n=7,958, Boys, n=8,183) | 12.0  (11.5, 12.5) | 14.0  (13.2, 15.8) | 10.2 (9.5,10.9) | 12.0  (11.5, 12.5) | 14.2  (13.4, 15.0) | 10.1  (9.4, 10.8) |
|  |  | Normal (N=9,582)  (Girls, n=5,403, Boys, n=4,179) | 12.1  (11.5, 12.7) | 14.5  (13.6, 15.4) | 9.9  (9.0, 10.8) | 11.6  (11.0, 12.2) | 13.9  (13.0, 14.8) | 9.4  (8.5,10.3) |
|  |  | Slightly unhealthy (N=3,439)  (Girls, n=1,871, Boys, n=1,568) | 13.8  (12.7, 14.9) | 16.2  (14.5, 17.9) | 11.5  (9.9, 13.1) | 12.4  (11.3, 13.5) | 14.5  (12.9, 16.1) | 10.4  (8.9, 11.9) |
|  |  | Extremely unhealthy (N=204)  (Girls, n=85, Boys, n=119) | 17.4  (12.2, 22.6) | 14.4  (7.2, 21.6) | 18.8  (11.8, 25.8) | 15.0  (10.4, 19.6) | 12.2  (5.9, 18.5) | 16.2  (9.9, 22.5) |
|  | Perceived weight | Extremely underweight (N=1,877)  (Girls, n=576, Boys, n=1,301) | 12.0  (10.4, 13.6) | 15.3  (12.2, 18.4) | 9.7  (7.9, 11.5) | 11.8  (10.2, 13.4) | 15.1  (12.0, 18.2) | 9.4  (7.6. 11.2) |
|  |  | Slightly underweight (N=8,242)  (Girls, n=3,517, Boys, n=4,725) | 11.0  (10.2, 11.8) | 13.1  (11.8, 14.4) | 8.6  (7.7, 9.5) | 10.6  (9.8, 11.4) | 13.0  (11.7, 14.3) | 8.5  (7.6, 9.4) |
|  |  | Normal weight (N=13,419)  (Girls, n=7,408, Boys, n=6,011) | 12.0  (11.4, 12.6) | 14.4  (13.6, 15.2) | 9.8  (9.0, 10.6) | 12.1  (11.5, 12.7) | 14.4  (13.6, 15.2) | 9.8  (9.0, 10.6) |
|  |  | Overweight (N=11,352)  (Girls, n=5,630, Boys, n=5,722) | 12.9  (12.1, 13.7) | 14.8  (13.7, 15.9) | 11.5  (10.4, 12.6) | 12.6  (11.9, 13.3) | 14.4  (13.3, 15.5) | 11.4  (10.4, 12.4) |
|  |  | Obesity (N=2,100)  (Girls, n=833, Boys, n=1,267) | 17.1  (14.9, 19.3) | 17.8  (14.6, 21.0) | 16.3  (13.4, 19.2) | 16.6  (14.5, 18.7) | 17.0  (13.9, 20.1) | 16.2  (13.3, 19.1) |
|  | Perceived stress | Low (N=6,230)  (Girls, n=2,324, Boys, n=3,906) | 10.7  (9.9, 11.5) | 13.5  (12.1, 14.9) | 8.6  (7.7, 9.5) | 11.3  (10.4, 12.2) | 14.8  (13.2, 16.4) | 8.7  (7.8, 9.6) |
|  |  | Moderate (N=15,615)  (Girls, n=7,235, Boys, n=8,380) | 11.0  (10.5, 11.5) | 12.5  (11.7, 13.3) | 9.7  (9.1, 10.3) | 11.3  (10.8, 11.8) | 13.0  (12.2, 13.8) | 9.8  (9.2, 10.4) |
|  |  | High (N=15,145)  (Girls, n=8,405, Boys, n=6,740) | 14.2  (13.6, 14.8) | 16.4  (15.6, 17.2) | 12.2  (11.4, 13.0) | 13.4  (12.8, 14.0) | 15.3  (14.4, 16.2) | 11.8  (11.0, 12.6) |
|  | Loneliness | Low (N=16,859)  (Girls, n=6,929, Boys, n=9,930) | 11.0  (10.5, 11.5) | 12.3  (11.5, 13.1) | 9.8  (9.2, 10.4) | 11.6  (11.1, 12.1) | 12.9  (12.0, 13.8) | 10.2  (9.6, 10.8) |
|  |  | Moderate (N=13,751)  (Girls, n=7,228, Boys, n=6,523) | 12.5  (12.0, 13.0) | 14.8  (14.0, 15.6) | 10.5  (9.8, 11.2) | 12.2  (11.7, 12.7) | 14.6  (13.8, 15.4) | 10.0  (9.3, 10.7) |
|  |  | High (N=6,380)  (Girls, n=3,807, Boys, n=2,573) | 15.0  (14.1, 15.9) | 17.8  (16.6, 19.0) | 12.4  (11.1, 13.7) | 13.4  (12.5, 14.3) | 16.3  (15.0, 17.6) | 10.8  (9.5, 12.1) |
|  | Depression | No (N=26,547)  (Girls, n=12,050, Boys, n=14,497) | 11.3  (10.9, 11.7) | 13.2  (12.6, 13.8) | 9.7  (9.2, 10.2) | 11.6  (11.2, 12.0) | 13.7  (13.1, 14.3) | 9.8  (9.3, 10.3) |
|  |  | Yes (N=10,443)  (Girls, n=5,914, Boys, n=4,529) | 14.6  (13.9, 15.3) | 17.0  (16.0, 18.0) | 12.4  (11.4, 13.4) | 13.5  (12.8, 14.2) | 15.6  (14.6, 16.6) | 11.6  (10.6, 12.6) |
|  | Anxiety | Minimal (N=23,967)  (Girls, n=10,528, Boys, n=13,439) | 11.4  (11.0, 11.8) | 13.1  (12.5, 13.7) | 9.9  (9.4, 10.4) | 12.0  (11.5, 12.5) | 13.8  (13.1, 14.5) | 10.3  (9.8, 10.8) |
|  |  | Mild (N=8,723)  ( Girls, n=4,814, Boys, n=3,909) | 13.4  (12.7, 14.1) | 15.9  (14.9, 16.9) | 11.0  (10.0, 12.0) | 12.3  (11.6, 13.0) | 14.9  (13.9, 15.9) | 10.0  (9.0, 11.0) |
|  |  | Moderate (N=3,047)  (Girls, n=1,829, Boys, n=1,218) | 14.5  (13.3, 15.7) | 17.1  (15.4, 18.8) | 12.3  (10.5, 14.1) | 12.6  (11.4, 13.8) | 15.0  (13.3, 16.7) | 10.4  (8.7, 12.1) |
|  |  | Severe (N=1,253)  (Girls, n=793, Boys, n=460) | 15.0  (13.1, 16.9) | 17.7  (15.1, 20.3) | 12.3  (9.4, 15.2) | 12.5  (10.8, 14.2) | 15.0  (12.6, 17.4) | 10.1  (7.5,12.7) |
| Community | Nutrition education | No (N=19,707)  (Girls, n=9,677, Boys, n=10,030) | 12.2  (11.7, 12.7) | 14.4  (13.7, 15.1) | 10.3  (9.7, 10.9) | 12.1  (11.6, 12.6) | 14.3  (13.6, 15.0) | 10.2  (9.6, 10.8) |
|  |  | Yes (N=17,283)  (Girls, n=8,287, Boys, n=8,996) | 12.3  (11.8, 12.8) | 14.5  (13.7, 15.3) | 10.4  (9.8, 11.0) | 12.2  (11.7, 12.7) | 14.3  (13.5, 15.1) | 10.3  (9.7, 10.9) |
|  | Living arrangement | Family members (N=35,515)  (Girls, n=17,332, Boys, n=18,183) | 12.2  (11.8, 12.6) | 14.5  (14.0, 15.0) | 10.3  (9.8, 10.8) | 12.1  (11.7, 12.5) | 14.3  (13.8, 14.8) | 10.1  (9.6, 10.6) |
|  |  | Relatives (N=159)  (Girls, n=62, Boys, n=97) | 10.2  (5.5, 14.9) | 10.6  (3.1. 18.1) | 9.3  (3.6, 14.8) | 10.1  (5.5, 14.7) | 10.2  (2.9, 17.5) | 9.3  (3.7, 14.9) |
|  |  | Off campus (N=215)  (Girls, n=84, Boys, n=131) | 15.1  (10.4, 19.8) | 18.7  (10.4, 27.0) | 12.6  (7.1, 18.1) | 14.5  (9.9, 19.1)_ | 17.9  (9.9, 25.9) | 12.2  (6.8, 17.6) |
|  |  | On campus (N=1,025)  (Girls, n=451, Boys, n=574) | 13.8  (11.6, 16.0) | 14.5  (11.1, 17.9) | 13.4  (10.6, 16.2) | 13.6  (11.5, 15.7) | 14.2  (10.8, 17.6) | 13.2  (10.4, 16.0) |
| Societal | Socioeconomic status | High (N=4,349)  (Girls, n=1,818, Boys, n=2,531) | 14.8  (11.9, 17.3) | 14.6  (12.9, 16.3) | 11.6  (10.3, 12.9) | 13.7  (11.2, 16.2) | 14.7  (13.0, 16.4) | 11.5  (10.2, 12.8) |
|  |  | Medium (N=32,131)  (Girls, n=15,989, Boys, n=16,142) | 12.1  (11.7, 12.5) | 14.4  (13.9, 14.9) | 10.1  (9.6, 10.6) | 12.0  (11.6, 12.4) | 14.2  (13.7, 14.7) | 10.0  (9.5, 10.5) |
|  |  | Low (N=601)  (Girls, n=248, Boys, n=353) | 13.1  (12.0, 14.2) | 15.8  (11.5, 20.1) | 13.7  (10.3, 17.1) | 13.1  (12.0, 14.2) | 14.7  (10.6, 18.8) | 12.6  (9.4, 15.8) |

Note: In model 1, each value represents predictive probability(95% CI) adjusted for sociodemographic variables (i.e., age, school type, school grade, academic performance, parental maximum educational attainment), screen-time, physical activity, sleep, smoking status, alcohol use, and BMI. For each variable, the first category listed serves as the referent group

Model 2 simultaneously adjusts for all independent variables
